# A comprehensive platform for the analysis of ubiquitin-like protein modifications using *in vivo* biotinylation

**DOI:** 10.1038/srep40756

**Published:** 2017-01-18

**Authors:** Lucia Pirone, Wendy Xolalpa, Jón Otti Sigurðsson, Juanma Ramirez, Coralia Pérez, Monika González, Ainara Ruiz de Sabando, Félix Elortza, Manuel S. Rodriguez, Ugo Mayor, Jesper V. Olsen, Rosa Barrio, James D. Sutherland

**Affiliations:** 1 CIC bioGUNE, Bizkaia Technology Park, Building 801-A, 48160 DERIO, Bizkaia, Spain; 2 Novo Nordisk Foundation Center for Protein Research, Faculty of Health and Medical Sciences, University of Copenhagen, Blegdamsvej 3b, 2200 Copenhagen, Denmark; 3 Biochemistry and Molecular Biology Department, University of the Basque Country (UPV/EHU), 48940 Leioa, Spain; 4 ITAV, IPBS, Université de Toulouse, CNRS, UPS, 1 Place Pierre Potier Oncopole entrée B, BP 50624, 31106 Toulouse Cedex 1, France; 5 Ikerbasque, Basque Foundation for Science, Alameda Urquijo, 36-5 Plaza Bizkaia, 48011 Bilbao, Spain

## Abstract

Post-translational modification by ubiquitin and ubiquitin-like proteins (UbLs) is fundamental for maintaining protein homeostasis. Efficient isolation of UbL conjugates is hampered by multiple factors, including cost and specificity of reagents, removal of UbLs by proteases, distinguishing UbL conjugates from interactors, and low quantities of modified substrates. Here we describe bioUbLs, a comprehensive set of tools for studying modifications in *Drosophila* and mammals, based on multicistronic expression and *in vivo* biotinylation using the *E. coli* biotin protein ligase BirA. While the bioUbLs allow rapid validation of UbL conjugation for exogenous or endogenous proteins, the single vector approach can facilitate biotinylation of most proteins of interest. Purification under denaturing conditions inactivates deconjugating enzymes and stringent washes remove UbL interactors and non-specific background. We demonstrate the utility of the method in *Drosophila* cells and transgenic flies, identifying an extensive set of putative SUMOylated proteins in both cases. For mammalian cells, we show conjugation and localization for many different UbLs, with the identification of novel potential substrates for UFM1. Ease of use and the flexibility to modify existing vectors will make the bioUbL system a powerful complement to existing strategies for studying this important mode of protein regulation.

Protein homeostasis contributes to the natural equilibrium within the cells, and its disruption is often the cause or consequence of multiple diseases. The balance is achieved in many instances through post-translational modifications (PTMs) by ubiquitin (Ub) and ubiquitin-like (UbL) proteins, controlling the function, localization or stability of target proteins. Ub is a 76-aa polypeptide that can modify target proteins through the process of ubiquitination, the attachment of an activated Ub moiety through a C-terminal glycine to a lysine or selected other residues in the target substrate^1^. The process involves the activation of Ub by an E1 enzyme, the transfer of the active moiety to an E2 conjugating enzyme and, in many instances, the cooperation of an E3 ligase that binds both the E2-bound Ub and the substrate. Deubiquitinases (DUBs) can revert the modification, conferring flexibility and regulation to the process^2^. Although most commonly associated to protein degradation by the proteasome, more recently ubiquitination has been related to a wide variety of cellular processes, including protein trafficking and DNA repair among others. Ub itself can be ubiquitinated in any of its seven lysines or the initiating methionine, forming chains that can adopt different conformations. This constitutes a complex code that can lead modified proteins to different outcomes^3^.

Ub is the most conserved protein found in all eukaryotes. Approximately 20 proteins have been identified that are related to Ub, known as UbLs^4^,^5^. Some have recognizable sequence homology with Ub, while more divergent UbLs share similar structural features. All of them share the beta-grasp fold characteristic of Ub and all participate in processes similar to ubiquitination, suggesting a common ancestry to this family of proteins^4^. Among the UbLs, the one that shares the highest homology with Ub is NEDD8 (NEural precursor cell-expressed, Developmentally Downregulated 8). While thousands of Ub targets have been identified, the reported number of NEDDylated proteins is lower. Among those, the cullins are RING E3 ligases that link NEDDylation to the ubiquitination of a wide spectrum of targets that participate in many cellular processes^6^.

The Small Ub-like MOdifier, SUMO, is one of the most studied UbLs^7^. In vertebrates, there are five different SUMO genes, SUMO1-5. SUMO2 and SUMO3 are almost identical and share 50% identity with SUMO1. All SUMOs use the same E1 and E2 enzymes in the process of SUMOylation, and can participate in forming polySUMO or mixed Ub-SUMO chains. SUMO4 seems to be a pseudogene or it is not processed^8^,^9^, while the recently reported SUMO5 shows tissue-specificity and participates in nuclear body formation^10^. In yeast and *Drosophila* there is a single SUMO homologue, Smt3. SUMOylation has been related to transcriptional repression and response to cellular stresses such as DNA damage.

Other UbLs are less well characterized. ISG15 (Interferon-Stimulated Gene 15) is induced by interferons secreted by virus-infected cells and participates in the anti-viral immune response^11^. FAT10 (HLA-F Adjacent Transcript 10, also called UBD) is expressed in immune cells and can also be induced in other cell types by interferon gamma or TNF-alpha. FAT10 can mediate Ub-independent proteasomal degradation^12^,^13^. Neither ISG15 nor FAT10 are conserved in lower eukaryotes. Both are composed of two UbL modules, whereas other UbLs have a single module. The Ub-Fold Modifier-1, UFM1, is conserved in metazoans and plants. It has a role in erythroid and megakaryocyte development, homeostasis of the endoplasmic reticulum (ER) and vesicle trafficking^14^. ATG8 and ATG12 are involved in the regulation of autophagy. ATG8 is a lipid modifier that is conjugated to phosphatidylethanolamine and participates in autophagosome biogenesis. There are 6 ATG8 orthologues in humans, classified as GABARAP1-2 and MAP1LC3A-B. ATG12 is conjugated to at least one other protein in the outer part of the autophagosome membrane, where this complex acts as the E3 ligase for Atg8^15^. FAU (Finkel-Biskis-Reilly murine sarcoma virus, ubiquitously expressed) is synthesized as a fusion protein with the ribosomal protein S30. In macrophages, FAU inhibits lipopolysaccharide-induced signalling and phagocytosis, having an immunoregulatory role^16^. The Ub Related Modifier 1, URM1, is conserved in eukaryotes and its structure resembles that of ancient prokaryotic sulfur carriers. Sulfur donors involved in the biosynthetic pathway of thiamine (vitamin B1) and molybdopterin (MPT) in prokaryotes, ThiS and MoaD, have a certain homology with Ub and contain a beta-grasp fold. Therefore, URM1 might represent a bridge between the prokaryotic sulfur carriers and the eukaryotic UbLs^17^.

Given the variety of UbLs that exist, the different types of monotypic chains that they can potentially form, and that there can even exist hybrid chains containing different types of UbLs, a complex UbL code may underlie numerous biological and cellular processes. This has prompted researchers to develop different systems to isolate UbL-modified proteins using cultured cells, model organisms, and clinical tissue samples. Most approaches are modelled on those successfully used to study Ub, such as using epitope-tagged Ub (with 6xHIS or HA tags). Approaches to study endogenous Ub modifications include monoclonal antibodies directed against the di-Gly residual peptide found on Ub substrates after deubiquitination, or molecular traps formed from tandem Ub-binding domains (TUBEs)^18^,^19^.

Although more challenging due to its low abundance, SUMOylation has been extensively studied using similar approaches. Different single or double epitope tags fused to the N-terminus of SUMO have been used to enrich for conjugates. Examples include 6x-10xHIS, FLAG, Myc, 6xHIS-FLAG, 6xHIS-HA, FLAG-TEV and ProtA-TEV-CBP for use in cell culture as well as transgenic organisms^20^. DeSUMOylating activity is high in cell lysates, so chemical inhibitors or ideally denaturing conditions are used during capture of conjugates to preserve SUMOylation. Using exogenous tagged SUMO also allows the use of SUMO mutants that may be defective in chain formation (e.g. SUMO2-K11R) or optimized for mass spectrometry analysis (MS; e.g. SUMO2-T90R)^21^. To analyse endogenous SUMO conjugates, a method called PRISM has been developed for the identification of SUMOylation sites based on the chemical blockade of all free lysines, followed by treatment with a SUMO-specific protease and subsequent biotin-tagging of ‘freed’ lysines and identification by high-resolution MS^22^. Monoclonal antibodies with mapped epitopes in SUMO1 or SUMO2/3 allow the identification of SUMOylated proteins in endogenous conditions^23^. Finally, SUMO-traps have been developed to enrich and identify poly- or multi-monoSUMOylated proteins^24^,^25^. Identification of other UbL-modifications has been performed using exogenous epitope-tagged versions or using antibodies to detect endogenous substrates^26^,^27^,^28^,^29^,^30^,^31^.

Variations between these approaches can identify different targets due to diverse levels of stringency, solubility, expression levels, and antibody types and their affinities. Furthermore, antibody and molecular trap approaches are ideal but require additional expertise and expense for affinity resins. Using an *in vivo* biotinylation approach, it was previously demonstrated that a short N-terminal epitope (Bio or AviTag)^32^ on Ub is efficiently biotinylated in cultured cells and in living organisms (flies/mice) by using the *E. coli* BirA enzyme, and that biotinylated Ub (bioUb) is incorporated efficiently into substrates^33^,^34^. Lysis under denaturing conditions allows inactivation of UbL isopeptidases and the high affinity biotin-streptavidin interaction allows stringent washes, yielding pure UbL-modified proteins with almost no background from non-covalent interactors and non-specific contaminants. Here, using a modular multicistronic expression platform^35^, we have generated a set of tools to study each UbL modification under stringent conditions, suitable for MS or validation by Western blotting, in an accessible and affordable way. We applied the system to various UbL modifications in cell culture and *in vivo*, identifying known and potentially novel substrates. The bioUbL system can complement existing tools to study UbL modifications to understand their diversity, regulation, and roles in biological systems.

## Results and Discussion

### Generation of the bioUbL system in multicistronic vectors

When applying the BirA/Bio tagging system to other UbLs, we modelled the initial vectors on the bioUb-BirA fusion, which relied on the capacity of endogenous DUBs to process the tandem bioUb-BirA precursor for the separation of the bioUb and the biotinylating enzyme^33^,^34^. However, when this design was attempted for SUMO, a large proportion remained unprocessed, perhaps due to failure of endogenous SUMO proteases to access the cleavage site. We reasoned that co-expression of bioUbLs and BirA separately could improve this, using a multicistronic vector platform^35^. The Ac5-STABLE2 vector encodes three ORFs separated by viral 2A sequences, and were designed to allow modular exchange of the promoter and ORF elements. For the first ORF, we placed the biotinylation-target peptide (Bio) followed by sites to insert UbL cassettes. For the second ORF, we inserted BirA, encoding the enzyme that transfers biotin to the Bio epitope. To enhance expression, in some cases a human codon-optimized version was used (BirA^opt^)^36^. For the third ORF, we inserted cassettes to allow stable cell line selection (e.g. puro, puromycin resistant cassette) or selection with either drugs or fluorescence or both (GFPpuro, GFP-puro fusion protein). Also, in some cases the third ORF position was exchanged for the respective E2 conjugating enzyme for the UbL being studied, which enhanced the recovery of bioUbL conjugates. The convenience to express the respective E2 along with the bioUbL should be analysed with caution, as it will be discussed in the Conclusion.

**Figure 1:**
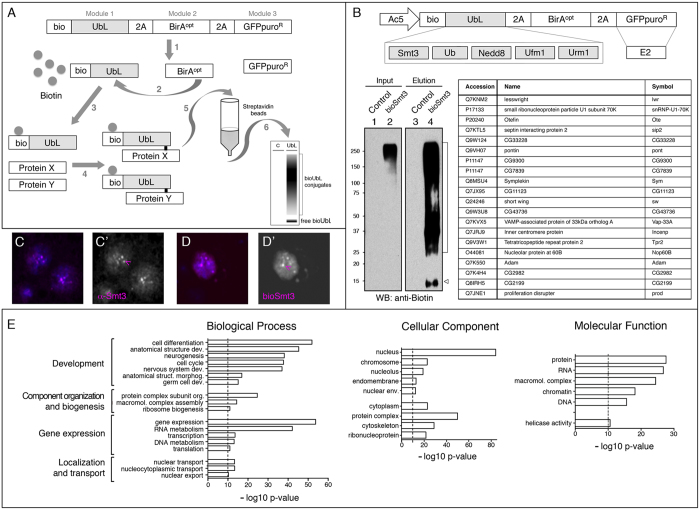
Isolation and identification of bioSmt3 conjugates in *Drosophila* cultured cells. (**A**) Schematic representation of the bioUbL strategy. See text for details. (**B**) Up: schematic representation of the bioUbL vectors for *Drosophila* cells. Left: the blot shows the enrichment in bioSmt3 conjugates in the elution panel using anti-biotin antibodies (lane 4; bracket). Arrowhead indicates free bioSmt3 in lane 4. Right: top twenty bioSmt3-modified proteins based on raw intensity. (**C**,**D**) bioSmt3 (*pAc510x-bioSmt3-Lwr*) visualized with fluorescently labeled streptavidin (**D**) localizes in a similar way than the endogenous Smt3 in S2R+ cells (**C**). Nuclei were marked with DAPI (blue). Green and red channels are shown independently in black and white (**C’**, **D’**). (**E**) GO analysis for biological process, cellular component and molecular function of the selected bioSmt3-conjugated protein set (n = 1054).

The resulting bioUbL-2A-BirA^opt^ (with puro, GFPpuro, or E2 conjugase; see Supplementary Data S1 for a summary of the vectors generated) is self-processed via 2A sequences (Fig. 1A, step 1), the BirA enzyme recognizes the Bio sequence (step 2), and transfers biotin to the lysine within the Bio sequence (step 3). The small C-terminal 2A remnant is removed during the activation step by endogenous UbL isopeptidases and, through action of the respective E2 conjugases and E3 ligases, the resulting bioUbL is incorporated into protein targets (X, Y, step 4). Modified proteins are purified by streptavidin-based affinity chromatography (step 5) and analysed by Western blot or MS (step 6). Importantly, lysis and pulldown are performed in denaturing conditions that inactivate UbL isopeptidases, selecting for recovery of true covalent UbL conjugates, while non-covalent UbL interactors are reduced. Since the components are genetically encoded, the system can be applied in both cell culture and transgenic organisms.

### bioUbL vectors for the analysis of PTMs in insect cells

As a proof of concept, to study UbLs different than Ub using the *in vivo* biotinylation approach, we chose to isolate and identify SUMOylated proteins in *Drosophila* cultured cells. *Drosophila* S2R+ cells were transfected with the *Ac5-bioSmt3-Lwr* vector or with *Ac5-FC-GP* vector as a control, which contains Flag-Cherry instead of bioSmt3. The vectors were appropriately expressed in cells (Supplementary Fig. S1). After pulldown we saw a notable enrichment of biotinylated proteins in the sample (Fig. 1B), as well as a prominent band corresponding to the properly processed bioSmt3 fusion.

*Drosophila* Smt3 localization is mainly nuclear and, as in the case of the SUMO mammalian homologs, Smt3 concentrates into discrete subnuclear domains (Fig. 1C). To confirm the localization of the biotin-conjugation system, we analysed bioSmt3 in the cells using streptavidin conjugated to a fluorescent dye (Fig. 1D). The comparison of the immunofluorescence experiments revealed that bioSmt3 localized in a way similar to the endogenous Smt3. In both cases, the proteins were mainly nuclear and localized in nuclear bodies, suggesting the proper subnuclear accumulation of the bioSUMOylated targets.

Compared to ubiquitination, modification by SUMO occurs at lower levels in cells in response to defined stimuli. For a particular target (which might be weakly expressed) only a small proportion of the cellular pool may get SUMOylated. We reasoned that supplying Lesswright (Lwr), the *Drosophila* homolog of SUMO E2 conjugase along with bioSmt3 (which must compete with endogenous Smt3) would allow detection of more conjugates while using less cellular lysate. To do this, we replaced the selectable marker *GFPpuro* by *lwr*. While we observed an appreciable increase in bioSmt3 conjugates (Supplementary Fig. S1), we caution that exogenous E2 expression might produce non-physiological SUMO modifications (see Conclusions). To further augment expression levels, we modified the Ac5 promoter to include 10 copies of the Upstream Activation Sequence, UAS, just before the transcriptional start site. This sequence is recognized by the yeast transcriptional activator Gal4. In the absence of Gal4, this modified Ac5 drives expression at lower levels than unmodified Ac5, but in the presence of Gal4, bioSmt3 levels were significantly higher. S2R+ cells were transfected with the *Ac510x-bioSmt3-Lwr* vector, or with *Ac510x-FC-Lwr* as a control, in presence or absence of the *pAc5-Gal4* vector. After the pulldown, total protein eluates were resolved on a SDS-PAGE and analysed by MS. We identified 1341 putative SUMOylated proteins, of which 1054, corresponding to 1042 different genes, were selected based on an 4:1 intensity ratio between the experimental samples and the controls (see Methods; Supplementary Data S2; Supplementary Fig. S2).

The Gene Ontology (GO) analysis revealed the enrichment of four biological process categories: development, component organization, gene expression or localization and transport (Fig. 1E, Supplementary Data S2). The majority of the proteins were localized in the nucleus, including proteins associated to the chromosomes, nucleolus or nuclear envelope. Cytoplasmic proteins were enriched for those classified as cytoskeletal and protein complex components. With respect to molecular function, we found enrichment of proteins that regulate gene expression, considered to be the main function of cellular SUMOylation, but also binding proteins (DNA, RNA, protein), underlining a role for SUMO in forming and regulating higher-order complexes.

Most of the identified proteins are functionally related, as revealed by the STRING analysis of interconnected networks (Supplementary Fig. S1). Cytoscape MCODE analysis showed 26 interaction clusters that confirm the implication in gene expression regulation of the identified SUMO targets (transcription, RNA processing and transport, splicing, translation), as well as vesicle-mediated transport, proteasome-mediated Ub-dependent protein catabolic process, or cell cycle (Supplementary Data S2).

549 proteins (52%) of the proteins found in S2 cells were found previously SUMOylated in other *Drosophila* experiments done using different systems and developmental stages: *Drosophila* embryos, 37%^37^ and immune challenged S2 cells, 46%^38^ (Supplementary Fig. S1). This is a substantial overlap considering the different experimental settings among studies (whole embryos *versus* cultured cells, unstimulated *versus* stimulated cells). Interestingly, this percentage of overlap is lower when we compare our list with the identified targets of bioUb in either whole embryos or adult eyes^39^, supporting the specificity of the bioSmt3 system.

Among the 20 bioSmt3-conjugates that showed highest intensities (all absent from control samples; Supplementary Data S2), some are involved in modulation of gene expression, involving transcription regulation (Pontin, Pont; CG7839; CG2982), translation initiation (Adam), polyadenylation (Symplekin, Sym), RNA binding/processing (CG11123; Nucleolar protein at 60B, Nop60B) and splicing (small ribonucleoprotein particle U1 subunit 70 K, snRNP-U1-70K). Others have been linked to nuclear functions, such as heterochromatic centromere and chromosomal segregation (Proliferation disrupter, Prod), mitosis (Inner centromere protein, Incenp) or components of the nuclear envelope (Otefin, Ote). In the cytoplasm, represented functions include transport and trafficking (Short wing, Sw; VAMP-associated protein of 33 kDa orthologue A, Vap-33A) and protein folding (Tetratricopeptide repeat protein 2, Tpr2). Among those 20, 18 were previously identified as targets of SUMOylation (Supplementary Data S2)^37^,^38^. In addition, the human homologues of 9 of them were also reported to be SUMOylated (UBE21, snRNP-U1-70K, Pont, CG7839, Sym, CG11123, Vap-33A, Nop60B and Adam), suggesting a conserved role for SUMOylation in the function of these proteins.

In addition to Smt3, the *Drosophila* genome encodes other UbL proteins. These include single homologs of NEDD8 (Nedd8), UFM1 (CG34191), and URM1 (CG33276). These genes were used to create bioUbL versions and expressed in S2R+ cells (Supplementary Data S1) and expression was confirmed in S2R+ cells. Together with bioUb-BirA (adapted from UAS-bioUb-BirA)^33^,^34^, these vectors will facilitate analysis of any Ub- or UbL-modification in *Drosophila* cells.

### Validation of bioSmt3-conjugates in *Drosophila* cells

**Figure 2:**
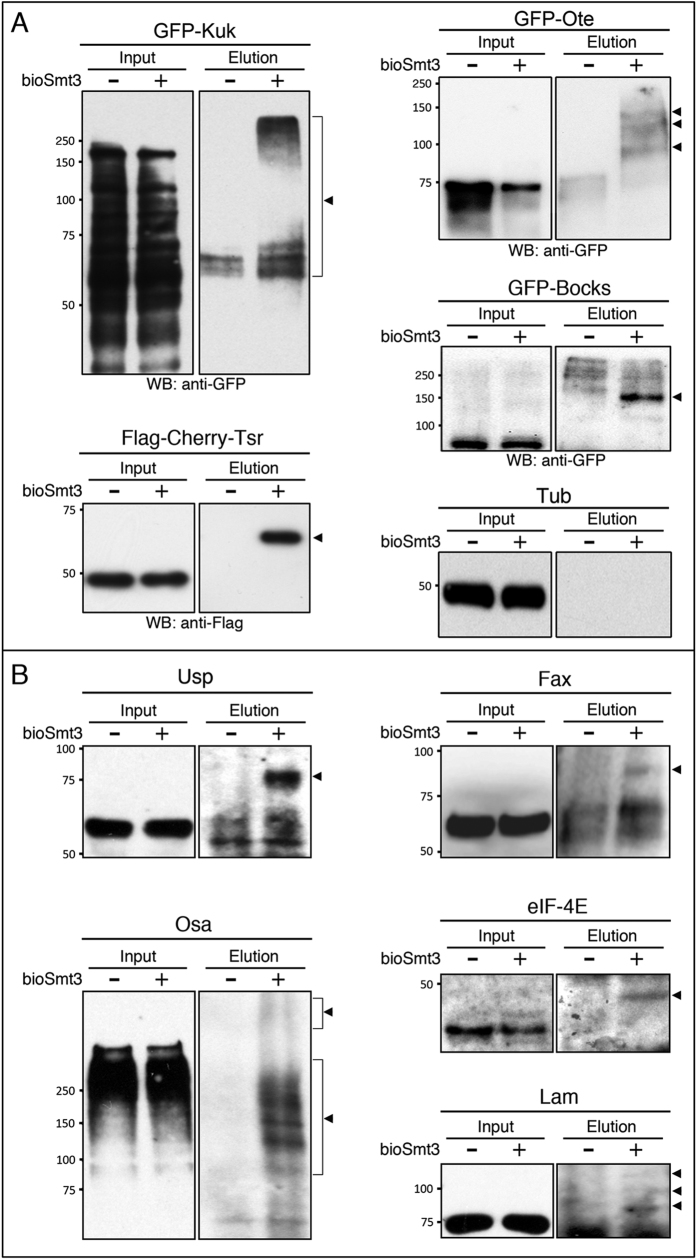
Validation of bioSmt3-modified proteins. (**A**) Anti-Flag or anti-GFP Western blot of pulldowns performed in S2R+ cells transfected respectively with *GFP-kuk, GFP-Ote, GFP-bocks* or *Flag-Cherry-tsr*, together with *pAc5-GAL4* and *pAc510x-bioSmt3-Lwr* (bioSmt3) (+) or *Ac510x-FC-Lwr* (−) as control. In the elution panels, arrowheads indicate the modified forms of each protein. Tubulin was used as a loading control. Molecular weight markers are shown to the left. (**B**) Western blot of pulldowns performed in S2R+ cells. Specific antibodies against endogenous proteins were used: Ultraspiracle (Usp), the Trithorax group protein Osa, Eukaryotic initiation factor 4E (eIF-4E), Failed axon connections (Fax) and Lamin (Lam). In elutions, arrowheads indicate the modified forms of the respective proteins. Molecular weight markers are shown to the left.

In order to validate our SUMOylation results, we chose Ote, Kugelkern (Kuk) and Bocksbeutel (Bocks), all components of the nuclear envelope, which is a structure enriched for SUMOylated proteins^40^,^41^,^42^ and is a described GO category represented by 34 MS hits in our list (Supplementary Data S2). Twinstar (Tsr) is the *Drosophila* orthologue of the actin destabilizing factor cofilin. Previous work has demonstrated SUMOylation of human cofilin^43^,^44^. Interestingly, we note that one of the identified SUMOylation sites of human cofilin (K13) overlaps with its reported nuclear export sequence (VIKVFNDMKV)^45^, which might have important consequences on localization for a small protein like Tsr (17 kDa). Tagged versions of these proteins (GFP- or FLAG-) were expressed in S2R+ cells, with and without bioSmt3. After the NeutrAvidin pulldowns, Western blots revealed the SUMOylated forms in the elution panels only in presence of bioSmt3 (arrowheads in Fig. 2A). Endogenous tubulin was used as a control for the input.

Despite the small proportion of SUMOylated fraction in the general pool of a given protein, we were able to validate bioSmt3 conjugation to some endogenous proteins by using available antibodies (Fig. 2B). Three of the validated proteins have been previously identified as SUMOylated proteins: Ultraspiracle (Usp), a nuclear receptor involved in steroid signalling^46^, the transcription factor Osa^37^,^47^, the intermediate filament Lamin (Lam, also a key nuclear envelope component)^38^ and the eukaryotic initiation factor 4E (eIF-4E)^48^. One of the validated proteins was not previously described to be SUMOylated in *Drosophila*, the Glutathione S-transferase involved in axonogenesis Failed axon connections (Fax), and thus is the first Fax homolog to be identified as a target of SUMOylation.

### *In vivo* application of bioSmt3 in transgenic *Drosophila*

**Figure 3:**
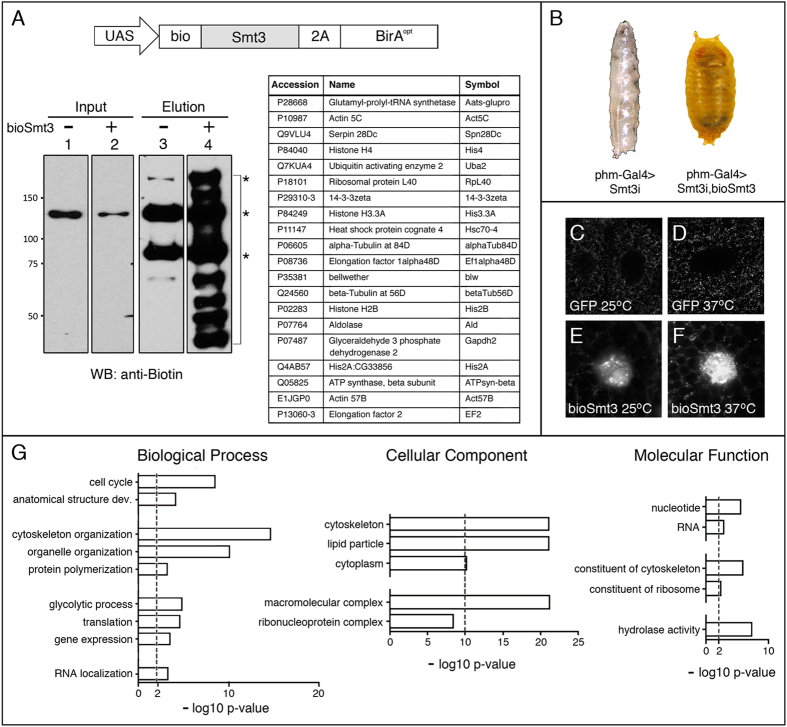
*In vivo* isolation and identification of bioSmt3 conjugates. (**A**) Up, schematic representation of the bioSmt3 vector used for *Drosophila* transgenesis. Down, the blot shows the enrichment of bioSmt3 conjugates in the elution panel using anti-biotin antibodies (lane 4, bracket; strain used: *hs-Gal4; UAS-bioSmt3*). Asterisks indicate the three known endogenously biotinylated proteins in *Drosophila*. (**B**) BioSmt3 can substitute for endogenous Smt3. Left, larvae silenced for *smt3* in the PG, arrested in development at the end of L3 larval stage. Right, the developmental arrest is rescued by the expression of bioSmt3 in the same genetic background. (**C**–**F**). Confocal pictures of cells of the salivary glands of larvae expressing bioSmt3 (**E**,**F**) or GFP (**C**,**D**). bioSmt3 is detected by fluorescently-labeled streptavidin. Heat treatment (37 °C) increased the bioSmt3-positive bodies. (**G**) GO analysis for biological process, cellular component and molecular function of the selected 140 bioSmt3-conjugated proteins.

We applied the bioUbL technology *in vivo* by generating transgenic flies expressing the transgene *bioSmt3* (Fig. 3A). To test the functionality of the bioSmt3 transgene *in vivo*, we analysed its capacity to rescue the silencing of endogenous *smt3* in steroidogenic tissues at larval stages. Tissue-specific knockdown of *smt3* in the prothorathic gland (PG) blocks development at the end of larval stages due to a reduction in the steroid levels^49^,^50^. This is achieved using a PG-specific GAL4 driver, together with *UAS-Smt3* RNAi (hereinafter called *smt3i*). Interestingly, by introducing an RNAi-resistant *bioSmt3* transgene in the *smt3i* background, larvae underwent normal metamorphosis and generated fertile adults (Fig. 3B). This indicates that bioSmt3 can complement at least this specific role for SUMOylation during development, which may also be the case in other tissues. Importantly, by increasing the bioSmt3/endogenous-Smt3 ratio, the chances of recovering rare bioSmt3 conjugates were increased.

We analysed the subcellular distribution of bioSmt3 conjugates *in vivo* by using fluorescent streptavidin in salivary glands from larvae expressing bioSmt3 (*hs-Gal4* > *UAS-smt3i, p-bioSmt3*) or from control larvae (*hs-Gal4* > *UAS-GFP*). Larval food was enriched with biotin. After using heat shock (hs) at 37 °C to both activate Gal4 and stimulate SUMOylation, we observed the accumulation of bioSmt3 in nuclear bodies, most likely conjugated to proteins (Fig. 3C–F). The biotinylated proteins showed a pattern similar to that observed for endogenous Smt3 using antibodies.

For the MS experiments, we collected larvae of *hs-Gal4* > *UAS-Smt3i, p-bioSmt3* or *hs-Gal4* > *UAS-Smt3i, pUAS-BirA*^*opt*^ genotypes. The E2 conjugase Lwr was not exogenously expressed in these experiments. To enrich the proportion of proliferating tissues, which exhibit a higher abundance of SUMOylated proteins^51^, we dissected and processed the anterior part of the larval body where the brain and most of the imaginal discs are located. After pulldown, we confirmed the enrichment of bioSmt3 conjugates (Fig. 3A). Protein eluates were resolved on SDS-PAGE and analysed by MS. We identified 251 proteins as putative SUMOylation targets *in vivo* (Supplementary Data S3, Supplementary Fig. S3), of which 140 were selected on the basis of a 4:1 intensity increase over controls.

The GO analysis (Fig. 3G) revealed common terms with the analysis in cultured cells, such as gene expression, translation or RNA, with a higher proportion of proteins localized in the cytoplasm, with the categories of lipid particle or cytoskeleton organization being strongly represented. The STRING analysis revealed a network of proteins, where 10 clusters could be identified (Supplementary Fig. S4, Supplementary Data S3). Among those, the most prominent is associated to the GO term translation, followed by others such as microtubule cytoskeleton, proteasome-mediated Ub-dependent protein catabolic process, chromatin assembly or disassembly or protein folding, among others. Some of the clusters reflect terms related to the methodology used in this experiment, such as the heat response used to induce expression and SUMOylation, or substrate adhesion, perhaps overrepresented due to the presence of the salivary glands in the lysed tissues. Approximately 50% of the proteins identified *in vivo* were also present in the list of bioSUMOylated proteins obtained from cultured cells and 34 or 37% were present in the lists of SUMOylated protein identified in *Drosophila* embryos or immune challenged S2 cells, respectively (Supplementary Fig. S4)^37^,^38^.

Among the 20 best-scoring bioSmt3 conjugates (Supplementary Data S3), we found parallels to our S2R+ dataset, for example proteins involved in different aspects of gene regulation, as chromatin and transcription (Histones His3.3A and His2B, CG7839), mRNA polyadenylation (Protein 1 of cleavage and polyadenylation factor 1, Pcf11) and translation (Eukaryotic initiation factor 4a, eIF-4a, and eIF4AIII), as well as cytoskeleton and protein trafficking (TER94, CG7852, Tubulins betaTub60D and betaTub85D, or Wings up A, WupA). 70% of these proteins (14/20) were identified as SUMOylated either previously or in this work in S2R+ cells. Also, the human homologues of many (17/20) have been independently identified as SUMO targets. CG42671, CG30069 and WupA were not previously identified as Smt3 targets. The human homolog of WupA the muscle-enriched TNNI2 (troponin I2, fast skeletal type) has never been identified as a possibly SUMOylated protein, which may reflect that most SUMO proteomics studies are done in cells rather than using whole tissues.

The notable difference in the number of putative SUMO conjugates when comparing the MS list obtained from S2R+ cells (1054) with the list obtained from larvae (140), is likely due to experimental differences. For example, cells yield better and more uniform lysates than tissues, biotin availability *in vivo* may be limiting and a lower quantity of *in vivo* material was processed compared to cells. Also, in flies we used RNAi to increase the bioSmt3/wild-type Smt3 ratio, while in S2R+ cells, we co-expressed the E2 conjugase *lwr* along with bioSmt3 to enhance the process of SUMO conjugation. These aspects might affect the yield of SUMOylated proteins in both cases and likely requires optimization for specific applications. Nonetheless, our results show that the bioSmt3 system can be efficiently applied to identify potential SUMOylated proteins in cultured cells and *in vivo* (Figs 1 and 3; Supplementary Data S2, S3).

### bioUbL vectors for the analysis of PTMs in mammalian cells

**Figure 4:**
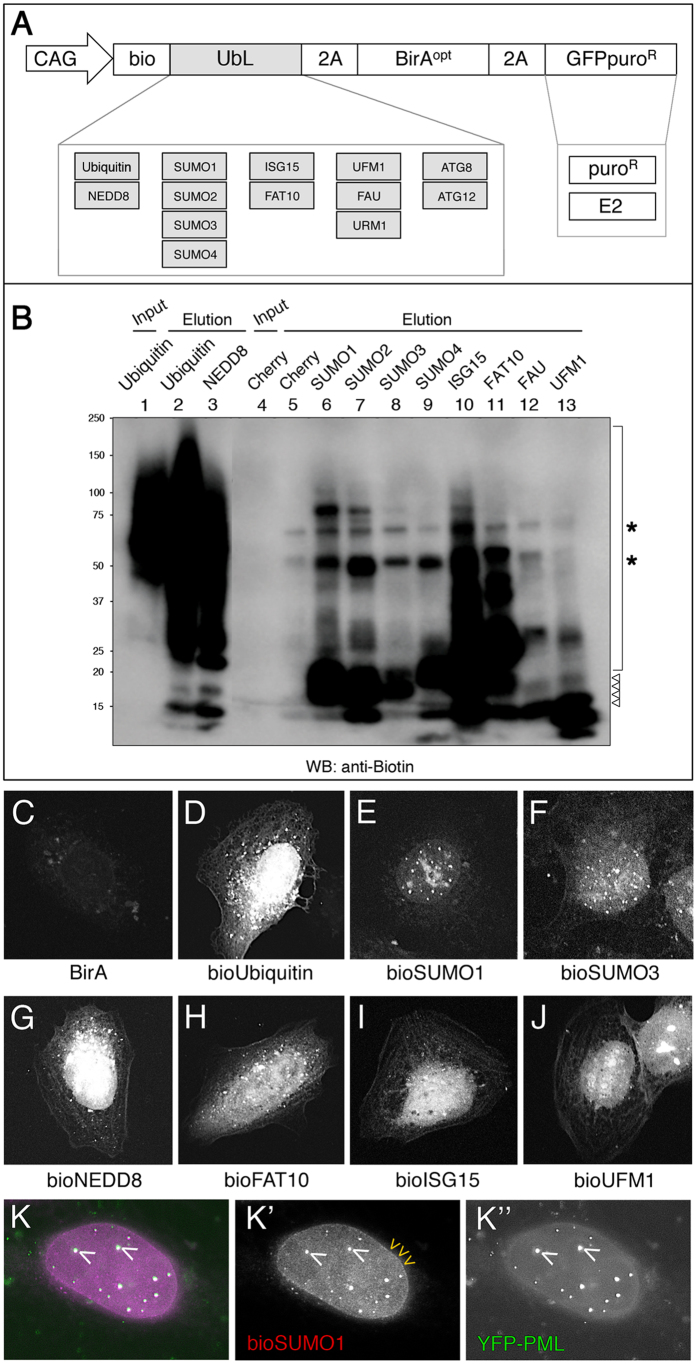
Isolation and localization of bioUbL-conjugates in mammalian cells. (**A**) Schematic representation of the bioUbL plasmid collection. (**B**) BioUbL-conjugates revealed by anti-biotin Western blot. Pulldowns were performed in parallel using HEK 293FT cells expressing the different bioUbLs. Conjugates are indicated with a bracket. Asterisks indicate endogenously biotinylated proteins. Arrowheads indicate free bioUbLs. Molecular weight markers are shown to the left. (**C**–**J**) Cellular distribution of different bioUbLs. U2OS cells transfected with plasmids expressing the indicated bioUbLs (**D**–**J**) or BirA alone as a control (**C**). Conjugates are visualized using fluorescently-labeled streptavidin. (**K**) White nuclear bodies (white arrowheads indicate two examples) reflect the colocalization of bioSUMO1 (purple) with YFP-PML (green). Yellow arrowheads indicate the localization of bioSUMO1 in the nuclear membrane. (**K’**) and (**K”**) show independently the green and purple channels in black and white, respectively.

Encouraged by the *Drosophila* results, we turned to mammalian cells to explore whether the system could work equally well and to broaden its applications. The modularity of the *pAc5-STABLE2-neo* vector facilitated the switch from the insect-specific promoter Ac5 to the versatile mammalian promoter CAG (Fig. 4A). We substituted the *Drosophila smt3* gene with the following mammalian UbLs: Ub, NEDD8, SUMO1-4, FAT10, ISG15, UFM1, FAU, and URM1 (Supplementary Data S1). All of these UbLs are thought to conjugate to protein targets through C-terminal glycine(s). Vectors expressing the autophagy-related ATG12 and ATG8 family (GABARAPs and LC3s) were also constructed, although these UbLs are thought to modify lipids more than proteins. Some variants of UbLs were also cloned (Supplementary Data S1). In most cases, the vectors carry a GFPpuro cassette to allow drug- or fluorescence-based selection, although other cassettes (resistance to puromycin, blasticidin, hygromycin) are possible. In some cases, the selection cassette is exchanged for the corresponding E2 enzyme to increase the efficiency of conjugation or address enzyme specificity (Fig. 4A). Using the modular architecture, other combinations could be envisioned, such as E1 activating enzymes, E3 ligases, substrates, and pathway modulators. Caution should be exercised since exogenous expression of E2 or indeed any other pathway enzyme may produce non-physiological modifications. Additional 2A-ORFs could also be inserted to expand the number of components for specific needs.

We tested the incorporation of the different bioUbLs to target proteins in HEK 293FT cells. The same number of cells were transfected in parallel with the different bioUbL constructs and NeutrAvidin pulldowns were analysed by anti-biotin Western blot (Fig. 4B). Although the cells were not submitted to additional stress (heat-shock, proteasome inhibition, etc), most of the tested bioUbLs were incorporated into target proteins. As expected, the relative amount of conjugated proteins varied depending on the modifier. For instance, bioUb, bioNEDD8 and bioISG15 showed the greatest level of incorporation, with some distinct conjugates being seen for bioSUMOs and bioFAT10. The bioUFM1 and bioFAU incorporation was negligible. This could be due to small amount of cells/lysate used, limiting expression levels of enzymes (E1/E2/E3) needed to incorporate the respective bioUbLs, or necessity of a particular stimulus to activate conjugation.

Since biotinylation occurs *in vivo*, we analysed the cellular distribution of the different bioUbLs by using fluorescent streptavidin (Fig. 4C–J). The different bioUbLs showed distinct localization patterns, suggesting that distribution in part may reflect localization of endogenous conjugates. Most of the UbLs are predominantly nuclear, although not exclusively, as seen with bioUb, bioNEDD8 or bioFAT10 (Fig. 4D,G,H). The bioSUMOs tested showed accumulation in nuclear bodies PML-positive, with bioSUMO1 showing further enrichment in the nuclear envelope (Fig. 4K); these structures are known to be enriched in SUMOylated proteins. The unique and characteristic patterns observed for each bioUbL suggests incorporation into a specific set of target proteins, further supporting the specificity of the bioUbL system.

### Assessment of bioSUMOs in human cells

**Figure 5:**
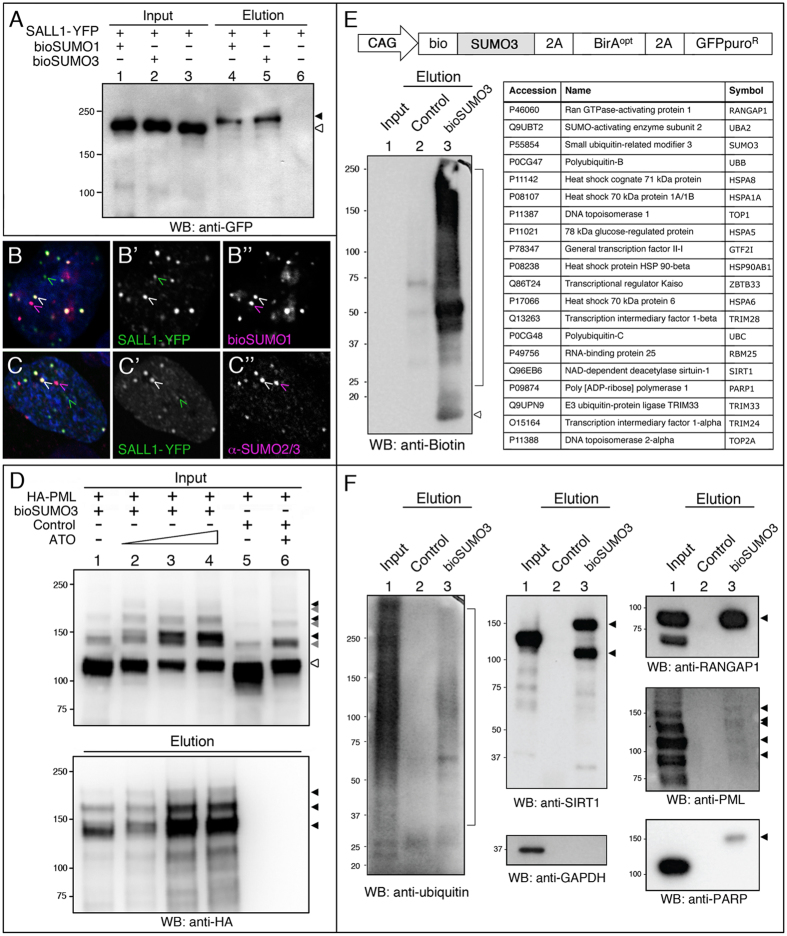
Isolation and identification of bioSUMO3-conjugates in mammalian cells. (**A**) Western blot of pulldowns from HEK 293FT cells showing that the transcription factor SALL1 fused to YFP was SUMOylated in presence (+) of bioSUMO1 (lane 4) or bioSUMO3 (lane 5; *bioSUMO1-BirA or bioSUMO3-BirA*, respectively). Black arrowhead indicates the modified SALL1-YFP in the elution panel (lanes 4, 5), which is shifted in comparison to the non-modified SALL1-YFP in the input panel (white arrowhead, lanes 1–3). Molecular weight markers are shown to the left. (**B**,**C**) Partial colocalization between SALL1-YFP (green) and bioSUMO1 (purple) (*bioSUMO1-BirA-UBC9*) in U2OS cells (**B**) or with endogenous SUMO2/3 (C, purple). White arrowheads indicate colocalization. Nuclei were stained with DAPI (blue). (**B’**,**C”**) Green and purple channels are shown independently in black and white. (**D**) SUMOylation of PML by bioSUMO3 (*bioSUMO3-BirA-GP*) increases after ATO treatment. bioSUMO3-modified PML (black arrowheads) can be detected by anti-HA Western blot in the input (upper panel, lanes 1–4) and the elution (lower panel, lanes 1–4; NeutrAvidin pulldown), while the level of the non-modified form of PML is reduced (input panel, lanes 1–6; white arrowhead). Note that modification of PML by endogenous SUMO is also visible in the input panel after ATO treatment (lanes 5 and 6, grey arrowheads). Control indicates cells transfected with *BirA-GP*. Molecular weight markers are shown to the left. (**E**) Up: schematic representation of the bioSUMO3 vector for mammalian cells. Left: the Western blot shows the enrichment in bioSUMO3 conjugates in the elution panel using anti-biotin antibodies (lane 3, bracket). Arrowhead indicates free bioSUMO3. Right: bioSUMO3-modified proteins identified by nLC MS/MS on Orbitrap. (**F**) Validation of bioSUMO3-modified proteins. Specific antibodies against endogenous proteins were used: Ub, SIRT1, RANGAP1, PML and PARP. GAPDH is shown as a control in the input panel. In the elution panels, arrowheads indicate the modified forms of the respective proteins and the bracket indicates the ubiquitinated proteins (lanes 3). Molecular weight markers are shown to the left.

To further evaluate the suitability of the system in mammalian cells, we chose to test SUMOylation of a specific target protein. The human transcription factor SALL1 was previously described to be SUMOylated *in vitro*^52^. By co-transfecting human SALL1 fused to YFP with bioSUMO1 or bioSUMO3 into HEK 293FT, we were able to confirm the SUMOylation of SALL1 in cells (Fig. 5A). We also observed the partial colocalization of the protein with bioSUMO1 (Fig. 5B) in a similar way than with the endogenous SUMO2/3 as revealed with the anti-SUMO antibodies (Fig. 5C).

PML SUMOylation is enhanced by the treatment with arsenic trioxide (ATO), triggering its polyubiquitination and subsequent degradation by the proteasome. Co-transfection of PML and bioSUMO3 was used to test the ATO response. As expected, after treatment we detected a decrease in the levels of unmodified PML in HEK 293FT cells (Fig. 5D, input panels, white arrowhead). In pulldowns, the levels of biotinylated PML increased, as expected from an increased incorporation of bioSUMO3 provoked by ATO (Fig. 5D, elution panels, black arrowheads; Control lanes in input panels show endogenous SUMOylation).

Besides testing for bioSUMOylation of particular proteins, we performed a small-scale MS experiment using HEK 293FT cells expressing bioSUMO3 (without E2 conjugase) or BirA alone. NeutrAvidin pulldowns were subjected to gel electrophoresis and stained with SYPRO-Ruby. Eleven prominent bands were excised and submitted for in-gel digestion and further identified by nLC MS/MS in Orbitrap. Twenty main proteins were identified in those bands, with all of them being previously described as SUMO targets, confirming the validity of the system (Fig. 5E). By Western blot analysis, we were able to observe bioSUMO3-modified forms for several of these targets, including Sirtuin 1 (SIRT1), Ran GTPase-activating protein 1 (RANGAP1), PML and Poly [ADP-ribose] polymerase 1 (PARP) (Fig. 5F). These experiments demonstrated that the bioSUMO system was sufficiently sensitive to detect the SUMOylated fraction of these proteins without stressing the cells. Interestingly, we identified Ub in the pilot MS experiment (Fig. 5E) and furthermore, we detected Ub in the pulldown lane of the Western blot (Fig. 5F), which suggests that either multiple PTMs (independent SUMO3 and Ub) or mixed-chain formation (SUMO3-Ub chains) can occur on substrates.

As seen with the *Drosophila* bioSmt3 vector, inclusion of the SUMO E2 enzyme UBC9 in the bioSUMO vectors led to a general increase the levels of modified targets. In the case of the bioNEDD8 vector, the inclusion of the corresponding E2 UBC12 both increased the level of modified proteins and changed the overall pattern (Supplementary Fig. S5). Specifically, the presence of UBC12 favours the lower molecular weight conjugates (two asterisks) in contrast to the higher molecular weight ones (one asterisk). Since NEDD8 overexpression has been reported to lead to misincorporation into non-habitual substrates by Ub pathway enzymes^53^, co-introduction of the NEDD8 E2 may suppress this effect. In addition, bioNEDDylation is drastically reduced in presence of MLN4924, an inhibitor of NEDD8 activating enzyme NAE1 (Supplementary Fig. S5), confirming that bioNEDD8 is processed by the same enzymes as the endogenous NEDD8. CUL3, a known NEDDylated protein, can be captured using the bioNEDD8 vector and detected using a specific antibody (Supplementary Fig. S5).

### Isolation and identification of bioUFM1 conjugates in human cells

**Figure 6:**
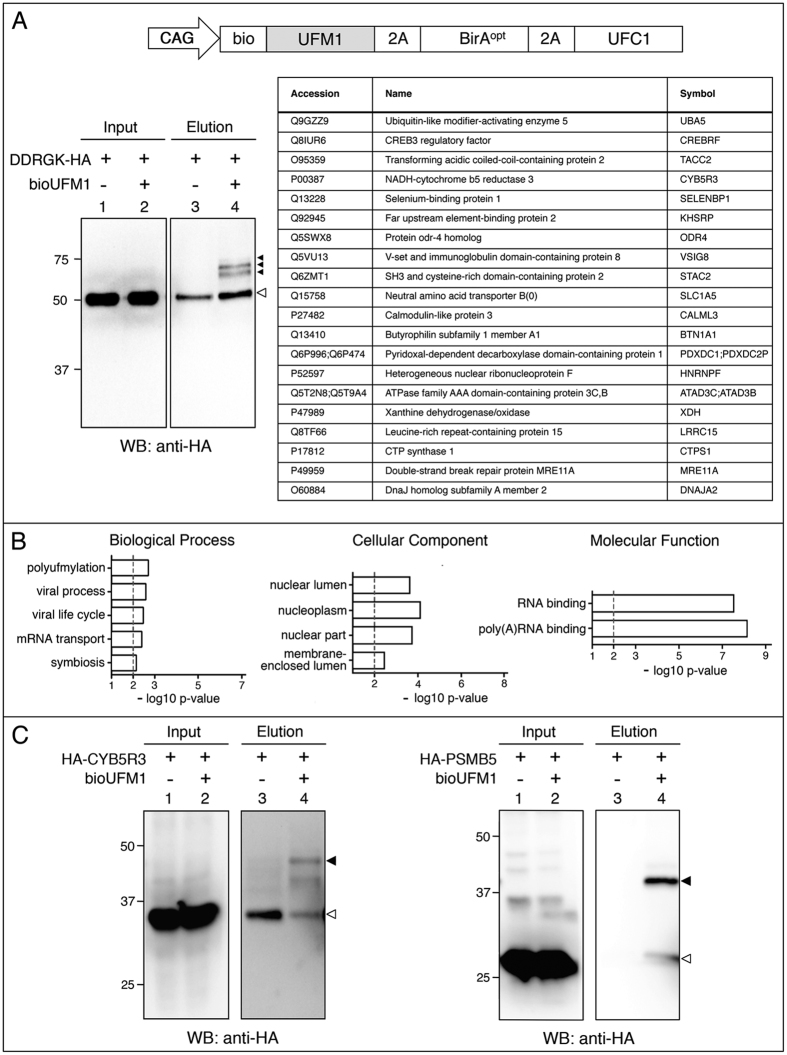
Isolation and identification of bioUFM1-conjugates in mammalian cells. (**A**) Up: schematic representation of the bioUFM1 vector for mammalian cells. Below, left: Validation of HA-DDRGK. Right, top twenty bioUFM1-modified proteins. (**B**) GO analysis for biological process, cellular component and molecular function of the selected bioUFM1-conjugated protein set (n = 82). (**C**) Validation of bioUFM1-modified proteins CYB5R3 and PSMB5 fused to HA tag. In the elution panels (lanes 3 and 4), black arrowheads indicate the modified forms of the respective proteins. Residual non-specific interactions of non-modified forms are indicated by white arrowheads. Molecular weight markers are shown to the left.

To further test the bioUbL approach in mammalian cells, we chose one of the less-studied UbLs, UFM1, to enrich for potential conjugates. To increase the efficiency of bioUFM1-conjugation, we co-expressed UFC1, the specific E2 enzyme involved in UFMylation (Fig. 6A). Few UFM1-conjugated proteins have been described, but perhaps the best characterized was DDRGK domain-containing protein 1 (DDRGK1, also known as UFBP1)^54^,^55^. A role for DDRGK1 as a co-factor of UFMylation has been postulated^30^, and knockout mice also display defects in embryonic erythroid development and adult hematopoiesis^56^. Using co-expression in HEK 293FT cells, we validated bioUFMylation of DDRGK1 (Fig. 6A).

To identify potential UFMylated proteins, we performed a larger-scale experiment using the bioUFM1/UFC1 construct in HEK 293FT cells. Since ER stress can upregulate the expression of UFM1 and UFMylation enzymes^55^, cells were treated with thapsagargin, a chemical inducer of ER stress (via inhibition of the ER Ca2+ ATPase pump). Neutravidin pulldowns were resolved by SDS-PAGE and analysed by MS. After normalization to remove background hits and curation, we identified 82 proteins that are potential UFMylated targets (see Methods; Supplementary Data S4, Supplementary Fig. S6). Among these hits, we did not detect peptides from DDRGK1, but this could be due to low expression levels of this protein in HEK 293FT.

Although UFMylation has been largely studied in the context of ER function, GO analysis showed that there was a significant enrichment in categories linked to RNA, such as mRNA transport (biological process) RNA, polyA-RNA binding (molecular function) or nucleoplasm (cellular component) (Fig. 6B; Supplementary Data S4). This is consistent with the observed nuclear localization of bioUFM1 in U2OS cells (Fig. 4J). The STRING analysis revealed an interconnected network of proteins, where 2 clusters were identified related with RNA splicing and ribonucleotide biosynthetic process (Supplementary Fig. S7, Supplementary Data S4). Of note, some proteins in our hit list are detected together in stable protein complexes (e.g. SLC1A5:CYB5R3, RTN4:ILF3, ILF3:SRRM2, PFAS:PPP6R3, SF3B1:ZNF326; DDX21:PARP1)^57^, or as yeast two-hybrid interactors (e.g. RTN4:SNX1)^58^, or as functional interactors (PARP1:MRE11)^59^,^60^. Since purification of bioUFM1 conjugates is under denaturing conditions and non-covalent interactions via UFM1 should be non-existent, we speculate that UFMylation may occur on multiple members of protein complexes, as it has been shown for SUMOylation and DNA repair complexes^61^. Interestingly, ODR4 (also called C1ORF27) is a homolog of *C. elegans* odr4, an ER-associated transmembrane protein found in complex with odr8, the *C. elegans* orthologue of UFSP2 (UFM1-specific protease 2)^62^.

Out of the 82 selected proteins, 47 have been reported as targets of SUMOylation and 45 as targets of ubiquitination (Supplementary Fig. S7, Supplementary Data S4). 32 are targets both of SUMOylation and ubiquitination. It is possible that these different UbL modifications might compete for the same target lysines or that they might take place at the same time. Peptides of Ub were also detected in the MS analysis, suggesting independent UFMylation and ubiquitination of the same substrate or, alternatively, the presence of UFM1-Ub mixed chains. Increases in scale and sensitivity are necessary to reveal the sites of UFM1 conjugation on substrates.

As expected, the co-expressed E2 UFC1 was identified. In pilot experiments that expressed only bioUFM1, endogenous UFC1 was identified (data not shown). Among the next 20 best-scoring bioUFM1 conjugates, we identified UBA5, the E1 enzyme that activates UFM1. Peptides from UFL1, the reported UFM1 E3 ligase, were not identified, but again could be due to expression levels and scale of the experimental setup. We found proteins involved in splicing and RNA trafficking (KHSRP- KH-type splicing regulatory protein, HNRNPF- heterogeneous nuclear ribonucleoprotein F), meiotic recombination and double-strand break (DSB) repair (MRE11A- MRE11 meiotic recombination 11 homolog A) and protein transport (SELENBP1- selenium binding protein 1).

We chose two MS hits to verify our results. Due to links of UFM1 to erythroid development and hematopoiesis, we chose cytochrome B5 reductase 3 (CYB5R3), since mutations can give rise to methemoglobinemia, characterized by the accumulation of blue-tinted methemoglobin in the blood, which gives the patients an unusual blue-tinted skin colour. Furthermore, CYB5R3 is ER-associated, specifically on the cytoplasmic side of the outer ER membrane^63^. A second interesting hit is PSMB5, one of the core beta subunits of the 20S proteasome. Mutations or overexpression of PSMB5 can lead to resistance to the proteasome inhibitor bortezomib, a chemotherapeutic agent for multiple myeloma and mantle cell lymphoma. Using bioUFM1 and exogenous UFC1 in HEK 293FT cells, we were able to detect UFMylated forms of both CYB5R3 and PSMB5, even without using ER stress conditions (Fig. 6C). Further functional assessment of the role of UFMylation for these targets will require additional effort.

Taken together, the bioUFM1 approach is an efficient method for the isolation and MS identification of potential UFMylated proteins in mammalian cells, as well as for the validation of specific targets. Due to the role of UFMylation in erythroid development uncovered through mouse genetic studies, exploration of these targets or discovery of new ones could be pursued in erythroleukemic or erythroid progenitor cell lines.

## Conclusions

Here we report the development of an efficient system to facilitate the study of potential UbL-modified proteins in both mammalian and *Drosophila* cells, as well as transgenic flies, that is suited for MS-based proteomics, validation and analysis of specific UbL-conjugated targets. This system represents multiple advantages with respect to other available UbL purification systems. The modular vectors represent a flexible platform to facilitate assembly of different elements into a single plasmid, even allowing *in vivo* biotinylation of non-UbL proteins of interest. Depending on the configuration, the vectors can be used for transient transfections, with FACS or drug selection allowing for enrichment, or for stable cell line generation. For bioUbLs, the possibility to co-express corresponding E2 conjugases or E3 ligases may enhance sensitivity and perhaps enrich for specific substrates of particular E2s or E3s. In the studies reported here, we have co-expressed E2 conjugases in the case of bioSmt3 in cells (but not in flies), and in the case of bioUFM1 (but not bioSUMO3). Expression of the E2 conjugases and/or E3 ligases, together with the bioUbL construct, might give rise to non-physiological modifications in cells, but this approach might facilitate the identification of novel targets below the sensitivity of other methods, or address substrate preferences for particular E3s or E2/E3 combinations. The co-expression strategy has been widely used in the literature for various UbLs, as it can be seen in some recent examples^30^,^64^,^65^,^66^,^67^,^68^,^69^,^70^. With caution in mind, we could not find specific reports showing that exogenous UBC9 or UFC1 drives unspecific modification of substrates. Related to this point, no gross developmental or physiological abnormalities were observed in mice that overexpress Ubc9 systemically, despite the overall increase in SUMOylation levels^71^. By overexpressing the bioUbL, we are likely saturating the existing endogenous E2 and only a certain amount of the bioUbL gets loaded (i.e. Lwr-bioSmt3). By supplying additional E2, cells will be able to make more Lwr-bioSmt3, which would lead to more SUMOylation and higher yield of lower abundance targets (so, more overall IDs in the MS analysis). UBC9 is involved in the selection of SUMO targets through its direct interaction with the SUMO consensus sites present in those targets; therefore, exogenous UBC9 expression might circumvent the need of E3 ligases or other stimulatory mechanisms (reviewed in ref. 7), but might not result in random SUMOylation of exposed lysines. However, the exogenous expression of E2/E3 enzymes might not reflect the physiological condition of a particular cell type/developmental stage/stress situation. The choice and convenience to supply exogenous E2 or not can be decided by researchers for their specific experimental settings, and secondary validations by complementary techniques is encouraged before embarking on detailed studies. All bioUbL vectors are provided without co-expression of E2s, while some of them co-express E2s, so that users can choose and compare when necessary.

To compare and contrast different approaches to UbL modifications, we highlight some advantages of the bioUbL system. The strong binding between biotin and streptavidin allows for purification under denaturing conditions, which maximizes solubility of the proteome and inactivates UbL “removal” enzymes (i.e. deubiquitinases, deSUMOylases, etc.). Likewise, strong binding allows high stringency washes that reduce non-specific background. Compared to the use of polyHis-tagged UbLs, only three endogenously biotinylated carboxylases are recovered as contaminants and are easily identified by MS. While antibody-based immunopurification of endogenous UbL-conjugates is ideal^23^, specific antibodies and commercial resins do not exist for all UbLs. Regarding the identification of UbL modification sites, use of antibodies that recognize branched-peptide remnants after trypsin digestion of UbL-modified proteins have been used to identify thousands of modification sites (Ub/NEDD8/ISG15^72^; modified SUMO^73^), but remains problematic due to expense, set-up, and specificity. Also, this approach is not yet available for all UbL modifications. Using SUMO as an example, identification of SUMO conjugation sites has been facilitated by introducing mutations close to the C-terminal di-Gly, allowing trypsin or LysC to generate short MS-compatible branched peptides^21^,^74^. The bioUbLs can be mutated as necessary to apply this strategy to SUMO and if needed, to other UbLs to facilitate conjugation site identification. In conjunction with antibodies to recognize an endogenous protein of interest, the bioUbLs can be used to quickly validate potential modifications by Western blotting. With co-expression of tagged proteins and appropriate scale-up, bioUbLs may also allow study of modification sites by MS, after single-step or two-step purifications. Subcellular localization of bioUbLs can be visualized using fluorescent streptavidin, although this application is limited in that one cannot distinguish between unconjugated and conjugated pools, nor localize *in situ* UbL modification of specific proteins. The application of bioUbLs in transgenic animals (as demonstrated for bioUB in *Drosophila* and mice)^33^,^34^,^39^ may allow tissue- or temporal-specific approaches to analyse UbL modifications. Use of the bioUbL system can provide either a global overview or a specific picture of the UbL modifications in most cell types and is compatible with drug treatments, environmental stress, and manipulations of other genes (overexpression, RNAi, CRISPR/Cas9 knockout or activation, etc.). We envision that it will complement existing techniques based on its high versatility, reliability and accessibility to most laboratories.

Taken together, these features make the bioUbL system a good choice for researchers interested in UbL modifications, either for validation when using proteins of interest or for explorative proteomics analysis.

## Methods

### Generation of vectors

All vectors (Supplementary Data S1) were based on *Ac5-STABLE2-neo* that contains three separate ORFs (Module1, Module 2, Module3; Fig. 1A) separated by 2A sequences^35^ (https://www.addgene.org/32426/). A codon-optimized version of BirA (BirA^opt^)^36^ was cloned into Module 2. In some cases, a C-terminal V5 epitope tag was added to facilitate detection (BirA^opt^V5). The Bio peptide (AviTag: MLNDIFEAQKIEWHE)^32^, was fused to a degenerated *Drosophila smt3* sequence resistant to RNAi (EMBL database accession number FN539078)^75^ to generate bioSmt3, and cloned into Module 1. A cassette encoding GFP-puromycin fusion (GFPpuro) or puromycin alone (puro) was cloned into Module 3. Additional bioUbL vectors were generated by substituting the *smt3* sequence. To generate the mammalian vectors, the *Drosophila* Actin5C (Ac5) promoter was substituted by the composite promoter *CAG*^76^. bioUbL vectors and cloning strategies are summarized in Supplementary Data S1.

For the generation of *CMV-SALL1-YFP*, the *SALL1* ORF was amplified by high-fidelity PCR and cloned into *EcoR*I-*Sal*I sites of pEYFP-N1. For *GFP-kuk, GFP-Ote* and *GFP-bocks*, the respective genes were amplified with stop codons from S2R+ cDNA by high-fidelity PCR and cloned into *Ac5-STABLE1-neo*. In the case of *Flag-Cherry-tsr*, the GFP module in *Ac5-STABLE2-neo* was substituted by the Tsr ORF amplified from S2R+ cDNA. For HA-CYB5R3, HA-PSMB5, and DDRGK1-2xHA, ORFs were amplified by high-fidelity PCR from 293FT or HeLa cDNA, and cloned into CB6-HA or CMV-2xHA. All vectors were checked by sequencing.

### Isolation of biotinylated substrates in mammalian and *Drosophila* cells

HEK 293FT cells (human embryonic kidney; Invitrogen) were maintained at 37 °C in DMEM (Dulbecco’s modified Eagle’s medium; Gibco) supplemented with 10% FBS, 1% penicillin/streptomycin (GIBCO). Transient transfections were performed using the calcium phosphate method in complete medium supplemented with biotin (50 μM). Cells were harvested 48–72 hours after transfection. For the isolation of bioSUMO3-conjugates coupled with MS, 10 × 10 cm dishes were transfected with *bioSUMO3-GP* or *FC-GP* as control. For the isolation of UFM1-conjugates coupled with LC-MS, 10 × 10 cm dishes were transfected with *bioUFM1-UFC1* or *BirA-puro*.

*Drosophila* S2R+ cells were obtained from DGRC (https://dgrc.cgb.indiana.edu) and cultured at 25 °C in *Drosophila* Schneider’s medium (Invitrogen) supplemented with 10% heat-inactivated fetal bovine serum (Gibco) and 1% penicillin/streptomycin (Gibco). Transfections were performed using calcium phosphate in 5 × 10 cm dishes with 2 μg of *pAC5-Gal4* (Addgene #24344), and 8 μg of *Ac510x-bioSmt3-Lwr* or *Ac510x-FC-Lwr* in complete medium supplemented with 50 μM biotin.

Transfected cells were collected after 48–72 hours (HEK 293FT) or 72–96 hours (S2R+), washed with phosphate buffered saline (PBS) and resuspended in lysis buffer [0.5 ml/10 cm dish; 8 M urea, 1% SDS, 50 mM N-ethylmaleimide, 1× protease inhibitor cocktail (Roche) in PBS]. Sonication was performed as needed to reduce sample viscosity. To reduce urea concentration, the samples were diluted by adding binding buffer (3 M urea, 1 M NaCl, 0.25% SDS; 0.5 volume for HEK 293FT, or 3 volumes for S2R+). Incubation was done using 100 μl suspension of high-capacity NeutrAvidin-agarose beads (Thermo Scientific) overnight at room temperature (RT). Washes were done according to Franco *et al*.^33^: 2× WB1, 3× WB2, 1× WB3, 3× WB4, 1× WB1, 1× WB5 and 3× WB6 [WB1: 8 M urea, 0.25% SDS in PBS; WB2: 6 M guanidine hydrochloride in PBS; WB3: 6.4 M urea, 1 M NaCl, 0.2% SDS in PBS (pre-warmed to 37 °C); WB4: 4 M urea, 1 M NaCl, 10% isopropanol, 10% ethanol, 0.2% SDS in PBS; WB5: 8 M urea, 1% SDS in PBS; WB6: 2% SDS in PBS]. Samples were eluted in 100 μl of 4× Laemmli sample buffer with 100 mM DTT by two cycles of heating (5 minutes; 99 °C), with vortexing in between. For MS analysis, the bead slurry was transferred to a Vivaclear Mini 0.8 μm PES filter (Sartorius) and spun to recover bead-free eluate.

### Isolation of biotinylated substrates *in vivo*

Flies were raised at 25 °C on standard *Drosophila* medium supplemented with biotin (50 μM). Strains: *w;pUAS-BirA*^33^; *w;UAS-Smt3i*^50^; *y*^*1*^w^*^;P{Gal4-Hsp70.*PB}31-1*/*CyO;TM6B*,*Tb*+ (called here *hs-Gal4*), Bloomington *Drosophila* Stock Center, BDSC#1822; *w;phm-Gal4, UAS-mCD8::GFP*/*TM6B, Tb* (called here *phm-Gal4*)^77^; *w;UAS-nls-eGFP*/*CyO* (BDSC #4775); Information about other strains can be found in FlyBase (http://flybase.bio.indiana.edu). The plasmid *pJFRC81-bioSmt3* (backbone: Addgene #36432) was used for generating transgenic flies (BestGene).

Crosses between *w*;*UAS-Smt3i*,*p-bioSmt3* and *phm-Gal4* were performed at 25 °C. Crosses with *hs-Gal4* were done at 25 °C and *w;UAS-Smt3i*/+;*p-bioSmt3*/*hs-Gal4* larvae were selected at L2 instar. As controls, we used *w; UAS-Smt3i*/+;*pUAS-BirA*/*hs-Gal4* larvae. Approximately 200 larvae were collected per condition and three heat shock events were performed at 37 °C during 30 minutes each. Dissections were performed in cold PBS and the lysis was done by using 1 ml of lysis buffer [8 M urea, 1% SDS, 50 mM N-ethylmaleimide in PBS and protease inhibitor mixture (Roche)]. Larval tissues were homogenized, sonicated, and after clearing by centrifugation at 4 °C, the supernatants were used for NeutrAvidin pulldowns performed as described above for *Drosophila* cells using 100 μl of suspension beads, using the same binding buffer dilution and washing/elution protocol.

### Mass Spectrometry

Pulldown elutions were separated by SDS-PAGE and stained with Brilliant Blue G-Colloidal Concentrate (Sigma) according to manufacturer’s instructions. Gel bands were excised from the whole gel lane, destained and proteins were in-gel digested with trypsin (sequencing grade, Promega) overnight as previously described^78^. The resulting peptide mixtures were extracted, desalted, and concentrated prior to online nanoLC-MS/MS analysis using and EASY nLC1000 system (Proxeon) connected to a Q-Exactive orbitrap (Thermo Scientific) through a nanoelectrospray ion source^79^. All raw LC-MS files were processed with MaxQuant software (version 1.4.0.3, www.maxquant.org) and search against species-specific Uniprot protein sequence databases and common contaminants using the Andromeda peptide search engine with a false discovery rate of 0.01 at both peptide and protein level. In the case of bioSUMO3, a nano Acquity (Waters) coupled on-line to an Orbitrap-XL (ThermoFisher) was used. Orbitrap raw MS files were processed with Proteome Discoverer (version 1.2) and searched with Mascot (Matrix Science).

The lists of proteins identified by MS were analysed as follows. First, contaminants and proteins identified by only one peptide were eliminated. Then, only those proteins with at least four-fold higher iBAQ values (label-free intensity-based absolute protein quantification) in the experiment samples *versus* the controls were considered as positive hits. Calculation was done taking into account a baseline for the control, which corresponds to the minimum iBAQ values registered in each control of every experiment.

Significantly enriched Gene Ontology annotation terms were determined using VLAD (http://proto.informatics.jax.org/prototypes/vlad-1.0.3/), InnateDB^80^ and G:profiler^81^. Graphical representation in Supplementary Figs S2, S3 and S6 was done using Prism GraphPad and eulerApe v3^82^. Network association analysis was done using STRING database^83^ and analysed using Cytoscape MCODE clusters and Cluster maker Visualizations^84^,^85^.

### Western Blotting

Input, flowthrough (FT) and elution samples were collected at different steps of the pulldown protocol. Elution samples were prepared as described above. Input and FT samples were adjusted to 4× Laemmli buffer. After boiling, proteins were separated by SDS-PAGE (BioRad) and blotted using wet transfer to PVDF membranes (Millipore). Membranes were blocked in 1× PBS with 0.1% Tween-20 (PBS-T) and 5% non-fat dry milk for 1 hour. When anti-biotin antibody was used, 1× Casein Blocking Buffer in PBS (Sigma #B6429) was used. Primary antibodies were used in blocking buffer (1 hour at RT, or overnight at 4 °C) as follows: mouse monoclonal anti-Usp (1:200)^86^; mouse monoclonal anti-Osa (1:50, DSHB); mouse monoclonal anti-Lamin Dm0 (1:500, DSHB #ADL84.12); rabbit polyclonal anti-Fax (1:1000)^87^; rabbit polyclonal anti-eIF4E (1:1000)^88^; rabbit polyclonal anti-SIRT1 (1:1000, Cell Signaling Technology #D739); mouse monoclonal anti-RanGAP1C-5 (1:1000, SantaCruz #sc-28322); rabbit polyclonal anti-PARP (1:1000, Cell Signaling Technology #9542); rabbit polyclonal anti-PML (1:1000, Bethyl Laboratories); rabbit polyclonal anti-Ub (1:1000, Sigma #U5379); rabbit polyclonal anti-Cullin 3 (1:1000, Cell Signaling Technology #2759); mouse monoclonal anti-Flag M2 (1:1000, Sigma #F3165); mouse monoclonal anti-GFP (1:1000, Roche #11814460001); mouse monoclonal anti-HA (1:1000, Sigma #H3663); mouse monoclonal anti-actin AC-74 (1:5000, Sigma #A2228); HRP conjugated anti-biotin (1:1000, Cell Signaling Technology #7075); HRP conjugated anti-tubulin (1:5000, Proteintech #66031); HRP conjugated anti-GAPDH (1:5000, Proteintech #60004).

After three washes with PBS-T, blots were incubated for one hour in blocking buffer with secondary antibodies: HRP-conjugated anti-mouse (1:5000, Jackson ImmunoResearch); HRP-conjugated anti-rabbit (1:5000, Jackson ImmunoResearch). Membranes were washed again three times in PBS-T and then developed using chemiluminescence with Clarity Western ECL substrate (Bio-Rad) or SuperSignal West Femto substrate (Thermo).

### Immunostainings

*Drosophila* S2R+ cells were transfected with calcium phosphate in 6 well-plates using 2 μg of *pAc5-bioSmt3-GP* or *pAC5-Gal4* (Addgene #24344). After 3 days, cells were placed on coverslips treated with concanavalin A (Sigma) and, once attached (4 hours), fixation was performed with 4% paraformaldehyde followed by permeabilization with 0.1% Triton X-100 in 1x PBS.

*Drosophila* larvae were dissected in 1x PBS and fixed for 20 minutes in 4% paraformaldehyde, washed three times in PBT (PBS, 0.3% Triton X-100) for 20 minutes, and blocked in PBT + 1% BSA for one hour.

U2OS cells were plated directly in 24-well plates with acid-washed coverslips and transfected using jetPEI (Polyplus) or Effectene (Qiagen) according to the manufacturer’s instructions. For the localization of different bioUbLs, 1 μg of *bioUbL-GP* or 1 μg of *BirA-puro* as control were used; for the localization of SALL1, 750 ng of *CMV-SALL1-YFP* and 250 ng of *bioSUMO1-2A-BirA-2A-UBC9* were used.

Antibodies and dyes: DAPI to label nuclei (1:15000, Sigma); Alexa Fluor 594 conjugated-streptavidin (1:200, Jackson ImmunoResearch) to visualize biotinylated proteins; mouse monoclonal anti-GFP (1:500, Roche); rabbit polyclonal anti-Smt3 (1:150)^89^; rabbit polyclonal anti-SUMO2/3 (1:100, Eurogentec #AV-SM23-0100). Secondary antibodies: anti-rabbit Alexa-568, anti-mouse Alexa-488 and anti-rabbit Alexa-488 (1:200, Molecular Probes). Samples were mounted in Prolong Gold antifade reagent (Molecular Probes). Confocal images were taken with a Leica DM IRE2 confocal microscope and images were processed using Adobe Photoshop CS5 software.

## Additional Information

**How to cite this article**: Pirone, L. *et al*. A comprehensive platform for the analysis of ubiquitin-like protein modifications using *in vivo* biotinylation. *Sci. Rep.* **7**, 40756; doi: 10.1038/srep40756 (2017).

**Publisher's note:** Springer Nature remains neutral with regard to jurisdictional claims in published maps and institutional affiliations.

## Supplementary Material

Supplementary Figures

Supplementary Dataset S1

Supplementary Dataset S2

Supplementary Dataset S3

Supplementary Dataset S4
