# Supplementary material for: A comprehensive platform for the analysis of ubiquitin-like protein modifications using in vivo biotinylation
Source: Sci Rep. 2017 Jan 18;7:40756. doi: 10.1038/srep40756 (PMC5241687; doi:10.1038/srep40756)
Supplement: Supplementary Figures [file srep40756-s1.pdf]

**SUPPLEMENTARY MATERIAL**

# A comprehensive platform for the analysis of ubiquitin-like protein modifications using *in vivo* biotinylation

Lucia Pirone[1], Wendy Xolalpa[1,2], Jón Otti Sigurðsson[3], Juanma Ramirez[4], Coralia Pérez[1], Monika González[1], Ainara Ruiz de Sabando[1], Félix Elortza[1], Manuel S. Rodriguez[5], Ugo Mayor[4,6], Jesper V. Olsen[3], Rosa Barrio[1*], James D. Sutherland[1*]

1. CIC bioGUNE, Bizkaia Technology Park, Building 801-A, 48160 DERIO, Bizkaia, Spain.
2. Present Address: Center of Research on Infectious Diseases, National Institute of Public Health, 62100 Cuernavaca, Morelos, Mexico.
3. Novo Nordisk Foundation Center for Protein Research, Faculty of Health and Medical Sciences, University of Copenhagen, Blegdamsvej 3b, 2200 Copenhagen, Denmark.
4. Biochemistry and Molecular Biology Department, University of the Basque Country (UPV/EHU), 48940 Leioa, Spain.
5. ITAV, IPBS, Université de Toulouse, CNRS, UPS, 1 Place Pierre Potier Oncopole entrée B, BP 50624, 31106 Toulouse Cedex 1, France.
6. Ikerbasque, Basque Foundation for Science, Alameda Urquijo, 36-5 Plaza Bizkaia, 48011 Bilbao, Spain.

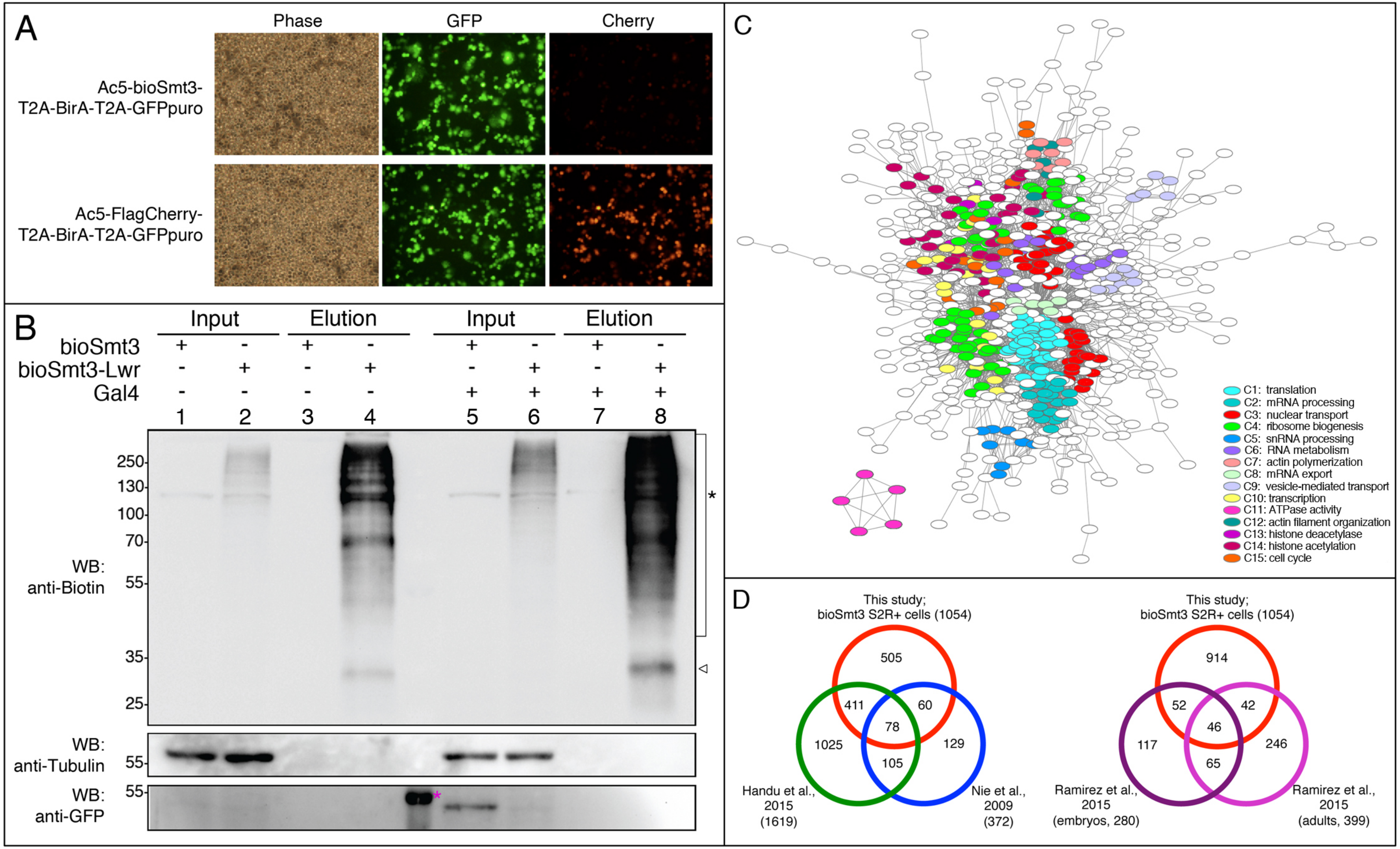
A
Phase
GFP
Cherry
Ac5-bioSmt3-
T2A-BirA-T2A-GFPpuro
Ac5-FlagCherry-
T2A-BirA-T2A-GFPpuro
B
Input
Elution
Input
Elution
bioSmt3
bioSmt3-Lwr
Gal4
1 2 3 4 5 6 7 8
250
130
100
70
55
35
25
WB:
anti-Biotin
WB:
anti-Tubulin
WB:
anti-GFP
C
C1: translation
C2: mRNA processing
C3: nuclear transport
C4: ribosome biogenesis
C5: snRNA processing
C6: RNA metabolism
C7: actin polymerization
C8: mRNA export
C9: vesicle-mediated transport
C10: transcription
C11: ATPase activity
C12: actin filament organization
C13: histone deacetylase
C14: histone acetylation
C15: cell cycle
D
This study;
bioSmt3 S2R+ cells (1054)
505
411
60
78
1025
129
105
Handu et al.,
2015
(1619)
Nie et al.,
2009
(372)
This study;
bioSmt3 S2R+ cells (1054)
914
52
42
46
117
246
65
Ramirez et al.,
2015
(embryos, 280)
Ramirez et al.,
2015
(adults, 399)

**Supplementary Figure S1. Expression of bioSmt3 in *Drosophila* cultured cells.** A. S2R+ cells transfected with *pAc5-bioSmt3-GP* or with *pAc5-FC-GP*. Both show GFP expression, while only the latter shows Cherry expression. B. Western blot showing the biotinylated proteins in the input (lanes 1-4) and elution (lanes 5-8) panels after pulldown of cells transfected with *Ac510x-bioSmt3-GP* (bioSmt3) or with *Ac510x-bioSmt3-Lwr* (bioSmt3-Lwr) in presence (+) or absence (-) of *pAc5-Gal4*. Tubulin is used as a loading control for the input. GFPpuro is shown in presence of Gal4 in the bioSmt3 lane. In the elution panels (lanes 4 and 8), arrowhead could indicate bioSmt3-Lwr complex and the bracket indicates the bioSmt3-conjugated proteins. Asterisk indicates an endogenously biotinylated protein. Molecular weight markers are shown to the left. Purple asterisk: non-specific reaction of the molecular weight marker with the anti-GFP antibody. C. Cytoscape analysis of the bioSmt3 network. Ovals represent the different SUMOylated proteins. Clusters of proteins detailed in Supplementary Data S2 are colored according to the legend. D. Venn diagrams showing the overlap of the bioSmt3-positive hits with other *Drosophila* SUMOylation screens (left) or with ubiquitinated proteins, identified using bioUb *in vivo* (right).

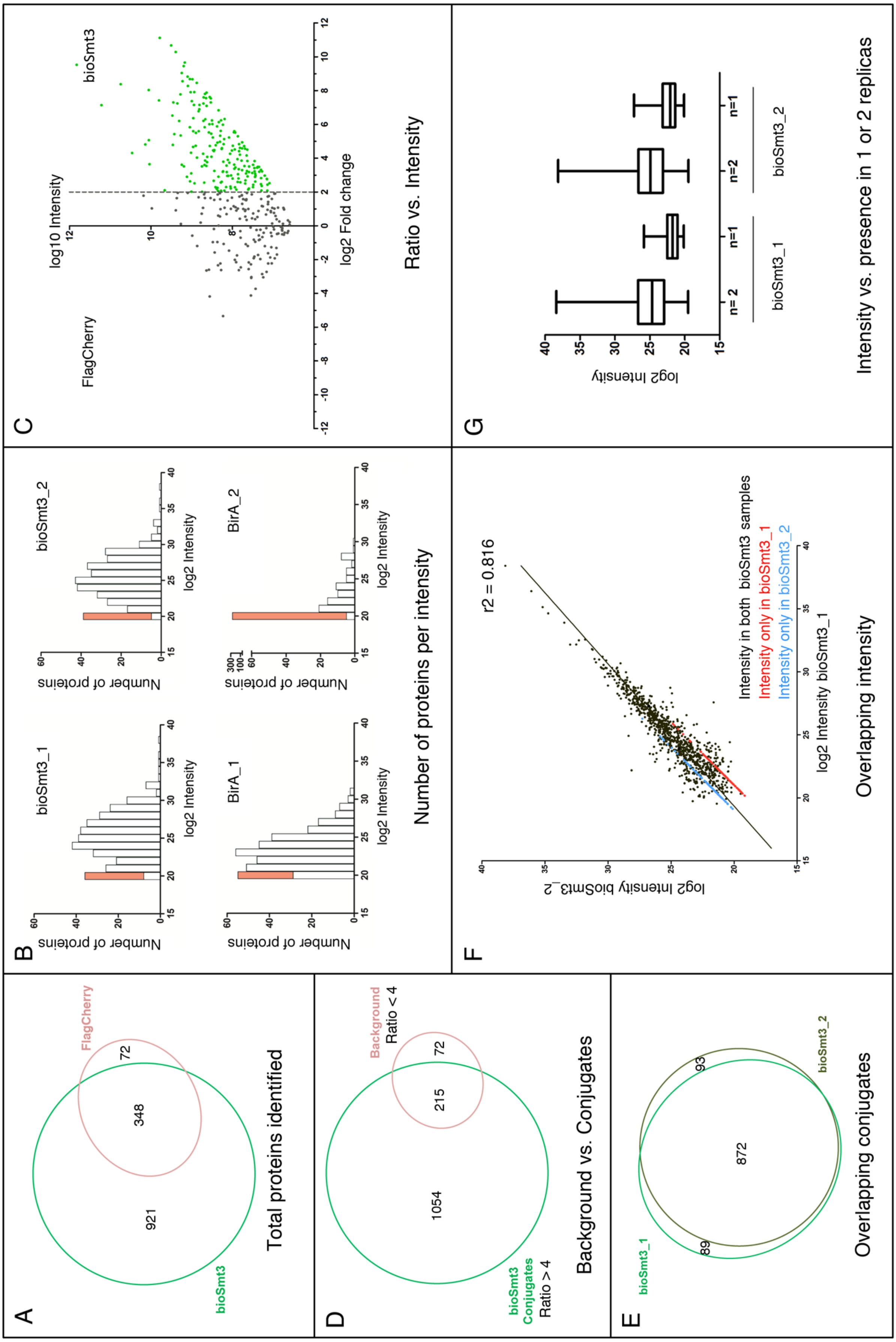
A
bioSmt3
FlagCherry
921
348
72
Total proteins identified
D
bioSmt3
Conjugates
Ratio > 4
Background
Ratio < 4
1054
215
72
Background vs. Conjugates
E
bioSmt3_1
bioSmt3_2
89
872
93
Overlapping conjugates
B
bioSmt3_1
bioSmt3_2
BirA_1
BirA_2
Number of proteins
log2 Intensity
Number of proteins per intensity
C
FlagCherry
bioSmt3
log10 Intensity
log2 Fold change
Ratio vs. Intensity
F
r2 = 0.816
log2 Intensity bioSmt3_2
log2 Intensity bioSmt3_1
Intensity in both bioSmt3 samples
Intensity only in bioSmt3_1
Intensity only in bioSmt3_2
Overlapping intensity
G
log2 Intensity
n= 2
n=1
n=2
n=1
bioSmt3_1
bioSmt3_2
Intensity vs. presence in 1 or 2 replicas

**Supplementary Figure S2. Analysis of the Mass Spectrometry data for bioSmt3 conjugates in *Drosophila* cells.** A. Venn diagram showing the overlap existing between the total number of proteins identified by MS in cells expressing *Ac510x-bioSmt3-Lwr* (bioSmt3) *versus* *Ac510x-FC-Lwr* (FlagCherry). B. Frequency histograms for bioSmt3 and FlagCherry (BirA) replicates indicating the number of proteins (*y* axis) found within different range of intensities (*x* axis) in MS analysis. Proteins for which no intensity was recorded were depicted in the lowest range of each group, colored with pink. Intensities are represented in log2 scale. C. SUMOylated proteins (green) were selected based on a 4-fold raw intensity ratio between the experimental samples (bioSmt3) and the controls (FlagCherry). Ratios with proteins for which no intensity was available were calculated using the lowest intensity reported in the MS analysis, although they were not represented in the graph. Fold changes (in log2 scale) *versus* the sum of the intensities (in log10 scale) detected among all FlagCherry and bioSmt3 analyses are plotted. D. Number of proteins classified as SUMO conjugates after subtraction of those considered background (i.e. bioSmt3/FlagCherry raw intensity ratio <4). E. Overlapping degree of the SUMO conjugates identified between the two replicates (bioSmt3_1 versus bioSmt3_2). F. Reproducibility between MS replicas. The Scatter plot represents the protein intensities (in log2 scale) obtained from replica 1 (bioSmt3_1) *versus* intensities from replica 2 (bioSmt3_2). The $r^2$ statistic was only calculated for proteins that have an intensity value in both replicas ($r^2 = 0.816$). Proteins with intensity only in one replica are also shown (red and blue dots). In this case missing intensities were estimated to allow graphical representation. G. More abundant proteins have better chances to be detected in both replicas than less abundant ones. Proteins detected in two replicas (n=2) are those found with bigger intensity (in log2 scale), while those detected in one replica (n=1) present lower intensity.

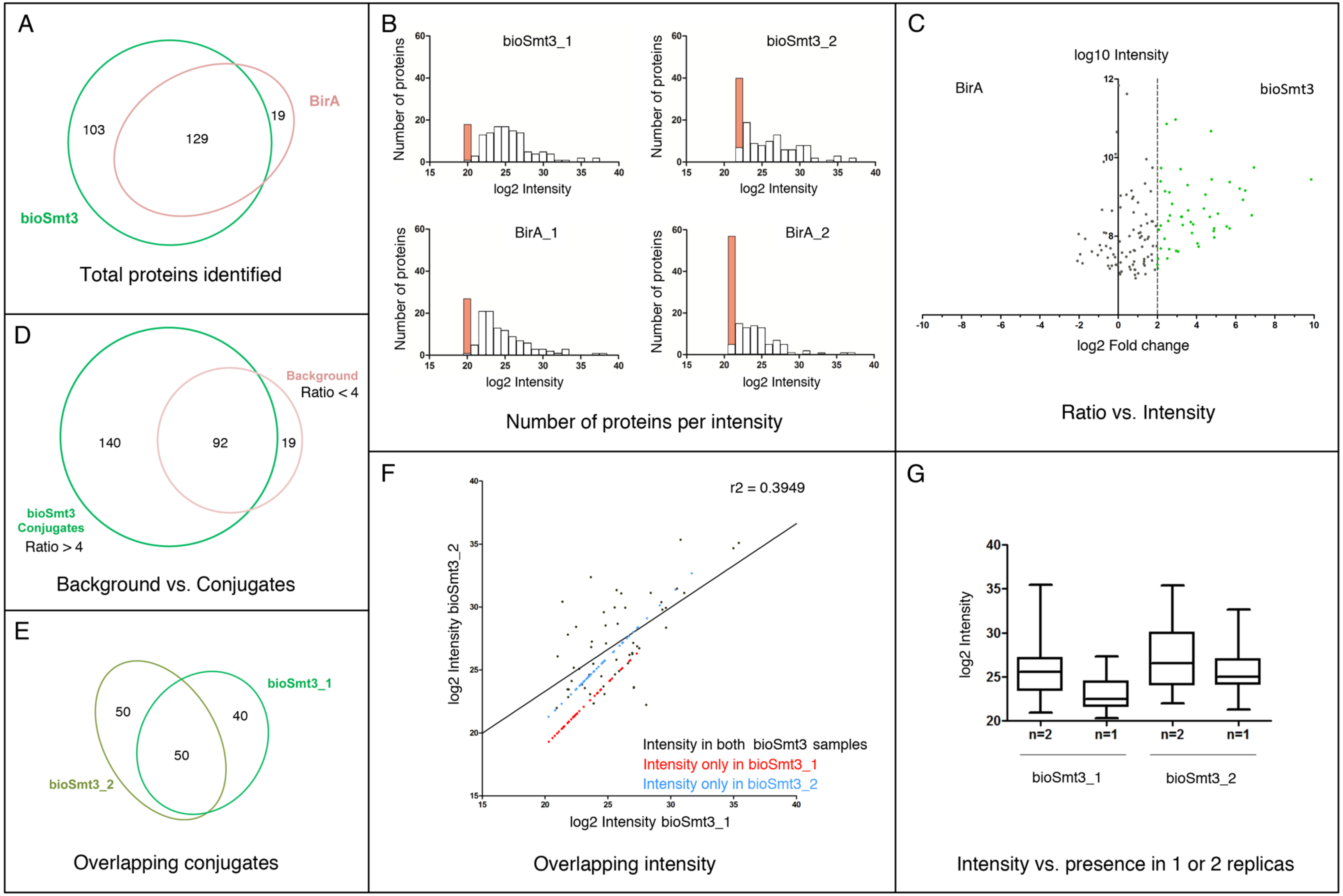
A
103
129
19
BirA
bioSmt3
Total proteins identified
B
bioSmt3_1
bioSmt3_2
BirA_1
BirA_2
Number of proteins
log2 Intensity
Number of proteins per intensity
C
log10 Intensity
BirA
bioSmt3
log2 Fold change
Ratio vs. Intensity
D
140
92
19
Background
Ratio < 4
bioSmt3
Conjugates
Ratio > 4
Background vs. Conjugates
E
50
50
40
bioSmt3_1
bioSmt3_2
Overlapping conjugates
F
r2 = 0.3949
log2 Intensity bioSmt3_2
log2 Intensity bioSmt3_1
Intensity in both bioSmt3 samples
Intensity only in bioSmt3_1
Intensity only in bioSmt3_2
Overlapping intensity
G
log2 Intensity
n=2
n=1
n=2
n=1
bioSmt3_1
bioSmt3_2
Intensity vs. presence in 1 or 2 replicas

**Supplementary Figure S3. Analysis of the Mass Spectrometry data for bioSmt3 conjugates *in vivo*.** A. Venn diagram showing the overlap existing between the total number of proteins identified by MS in larva expressing *pUAS-bioSmt3* (bioSmt3) *versus pUAS-BirA* (BirA) using the *hs-GAL4* driver. B. Frequency histograms for bioSmt3 and BirA replicates indicating the number of proteins (*y* axis) found within different range of intensities (*x* axis) in MS analysis. Proteins for which no intensity was recorded were depicted in the lowest range of each group, colored with pink. Intensities are represented in log2 scale. C. SUMOylated proteins (green) were selected based on a 4-fold raw intensity ratio between the experimental samples (bioSmt3) and the controls (BirA). Ratios with proteins for which no intensity was available were calculated using the lowest intensity reported in the MS analysis, although they were not represented in the graph. Fold changes (in log2 scale) *versus* the sum of the intensities (in log10 scale) detected among all BirA and bioSmt3 analyses are plotted. D. Number of proteins classified as bioSmt3 conjugates after subtraction of those considered background (i.e. bioSmt3/BirA raw intensity ratio <4). E. Overlapping degree of the bioSmt3 conjugates identified between the two replicates (bioSmt3_1 *versus* bioSmt3_2). F. Reproducibility between MS replicas. The Scatter plot represents the protein intensities (in log2 scale) obtained from replica 1 (bioSmt3_1) *versus* intensities from replica 2 (bioSmt3_2). The $r^2$ statistic was only calculated for proteins that have an intensity value in both replicas ($r^2 = 0.3949$). Proteins with intensity only in one replica were also plotted (red and blue dots). In this case missing intensities were estimated to allow graphical representation. G. More abundant proteins have better chances to be detected in both replicas than less abundant ones. Proteins detected in two replicas (n=2) are those found with bigger intensity (in $\log_2$ scale), while those detected in one replica (n=1) present lower intensity.

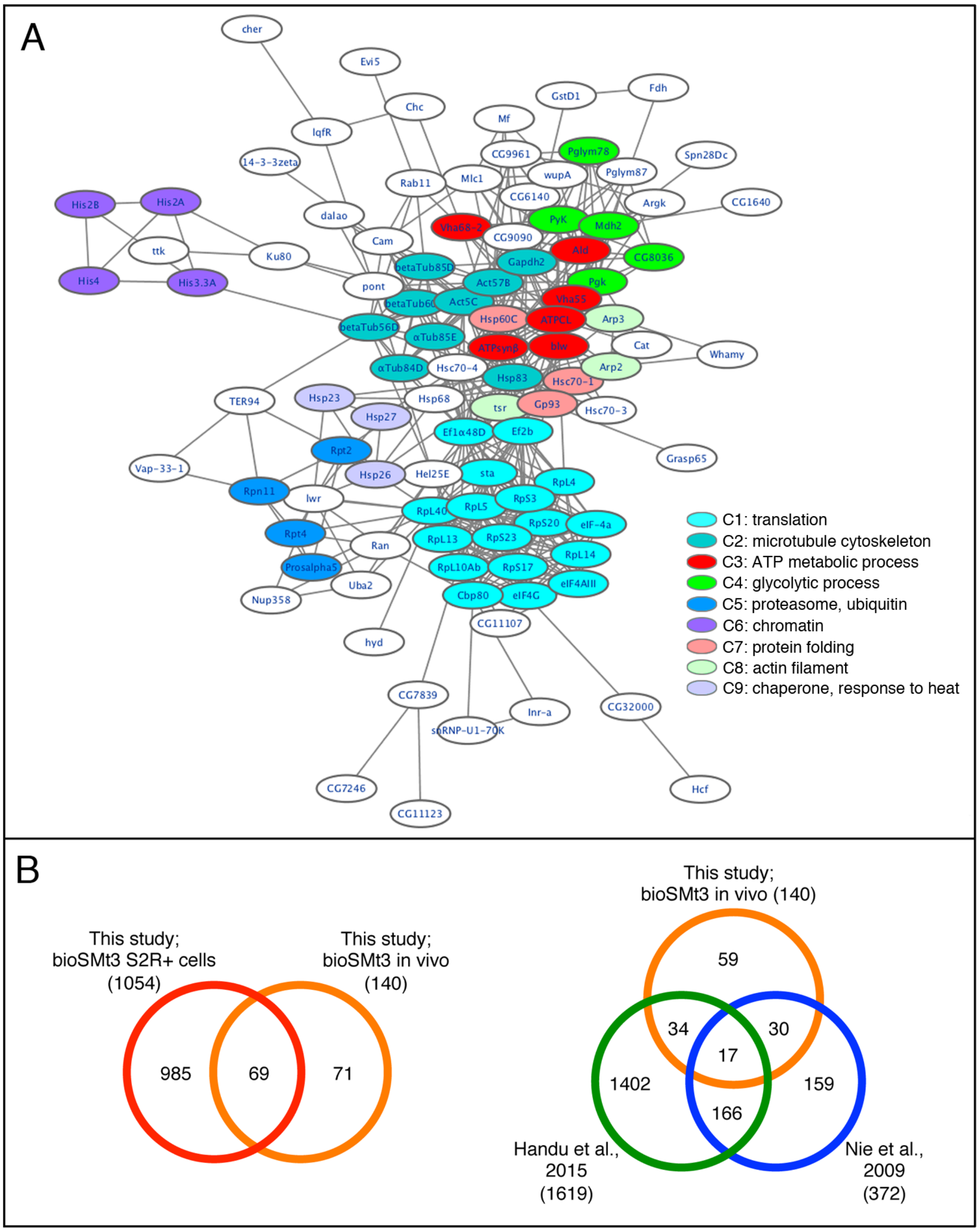


**Supplementary Figure S4. Networking analysis for bioSmt3 conjugates *in vivo*.** A. Cytoscape analysis of the bioSmt3 network. Ovals represent the different SUMOylated proteins. Clusters of proteins detailed in Supplementary Data S3 are colored according to the legend. B. Venn diagrams showing the overlap of the bioSmt3 positive hits *in vivo* with the bioSmt3 hits in cultured cells (left) or with the hits from other *Drosophila* screens (right).

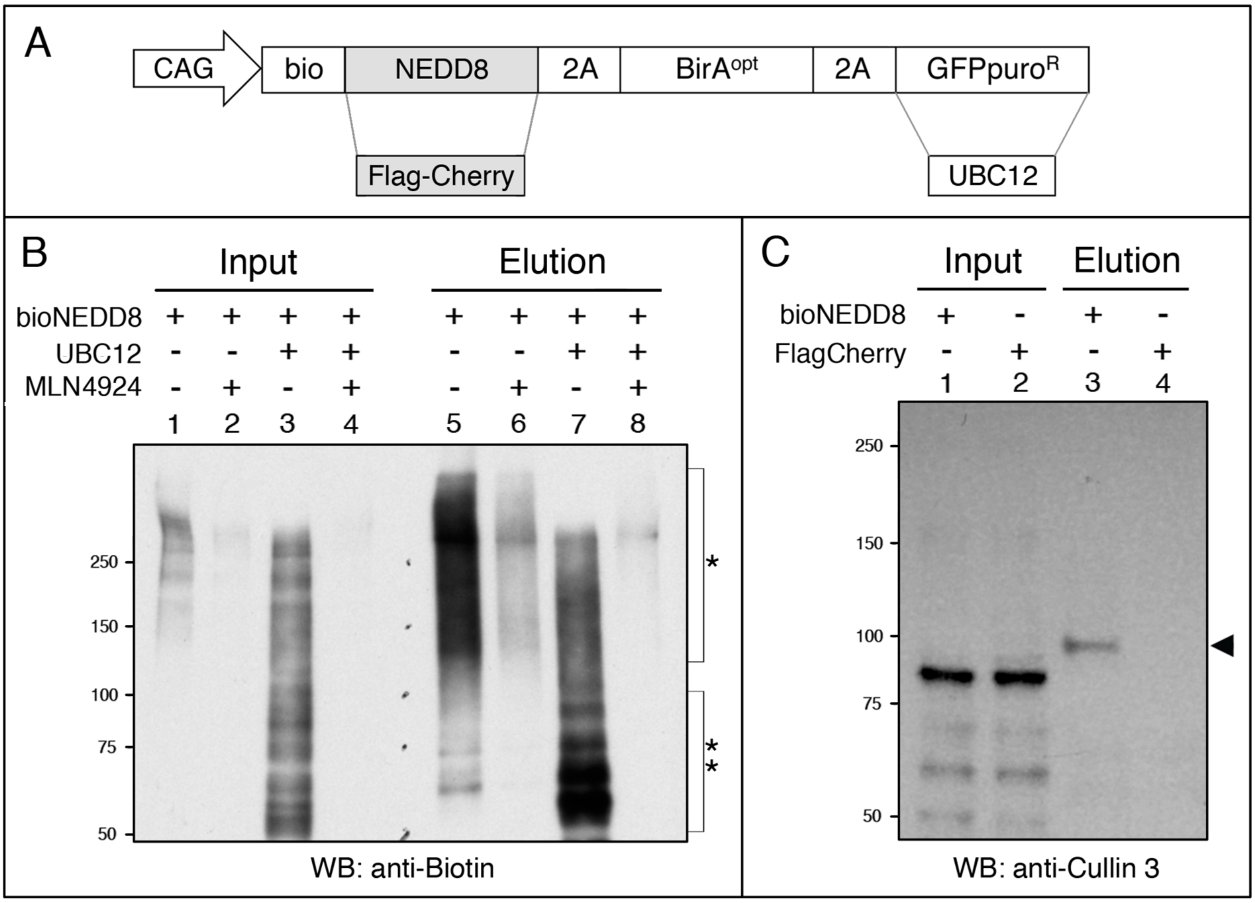


**Supplementary Figure S5. Isolation of bioNEDD8-conjugates in mammalian cells.** A. Schematic representation of the bioNEDD8 vector for mammalian cells. B. Western blot showing the biotinylated proteins in the input and elution panels after pulldown of cells transfected with *bioNEDD8-GP* (bioNEDD8) or with *bioNEDD8-UBC12* (UBC12) in presence (+) or absence (-) of MLN4924, an inhibitor the heterodimeric NEDD8 E1 enzyme NAE1. Brackets indicate high (one asterisk) or low (two asterisks) molecular weight bioNEDD8-conjugates (lanes 5 and 7). Molecular weight markers are shown to the left. C. bioNEDDylation of Cullin 3, a known target of NEDD8 in presence of *bioNEDD8-GP* (lane 3) but not of the negative control *FC-GP* (lane 4). In the elution panel, the black arrowhead indicates the modified form of Cullin 3. Molecular weight markers are shown to the left.

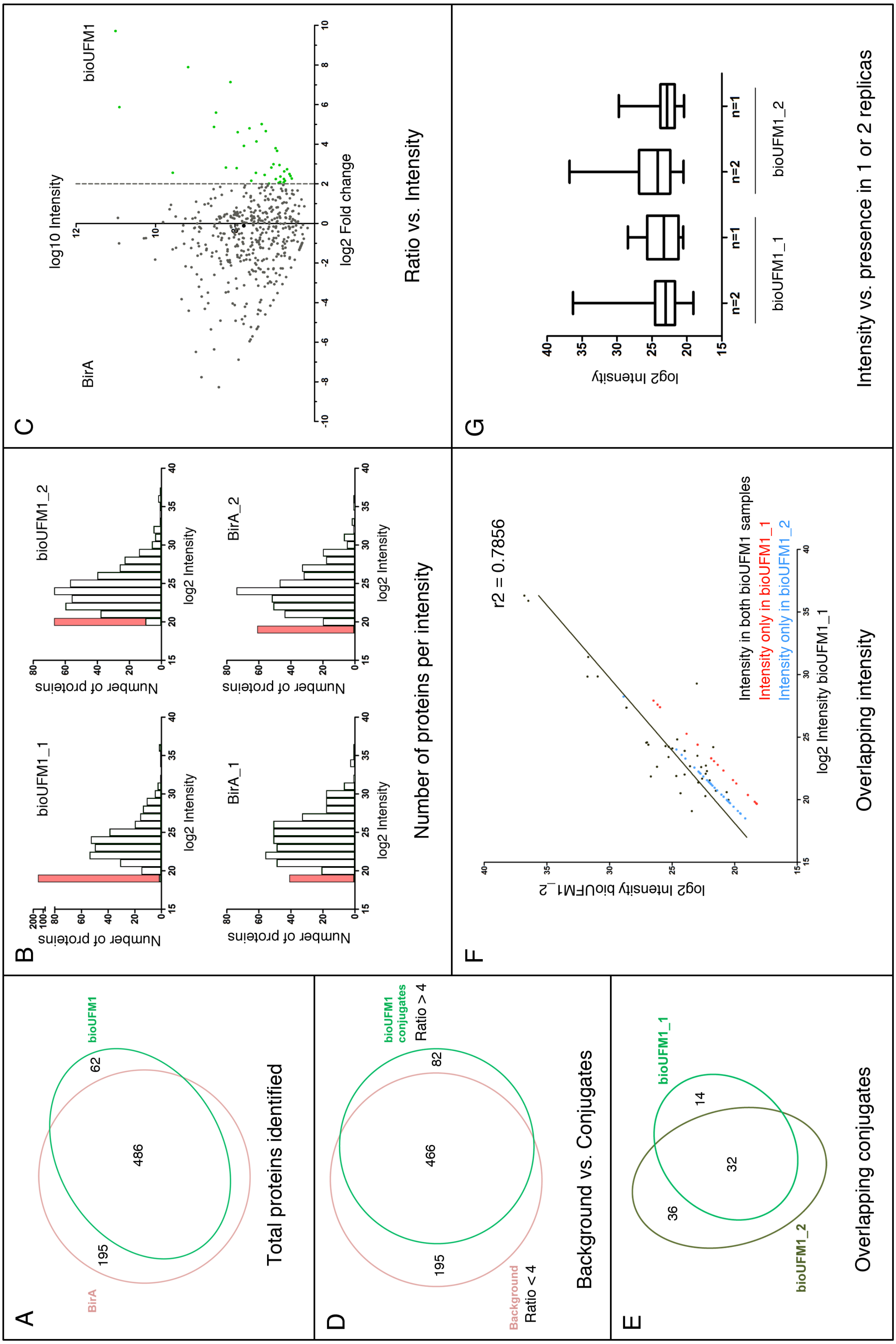
A
BirA
bioUFM1
195
486
62
Total proteins identified
B
bioUFM1_1
bioUFM1_2
BirA_1
BirA_2
Number of proteins
log2 Intensity
Number of proteins per intensity
C
BirA
bioUFM1
log10 Intensity
log2 Fold change
Ratio vs. Intensity
D
Background
Ratio < 4
bioUFM1 conjugates
Ratio > 4
195
466
82
Background vs. Conjugates
E
bioUFM1_1
bioUFM1_2
36
32
14
Overlapping conjugates
F
r2 = 0.7856
log2 Intensity bioUFM1_2
log2 Intensity bioUFM1_1
Intensity in both bioUFM1 samples
Intensity only in bioUFM1_1
Intensity only in bioUFM1_2
Overlapping intensity
G
log2 Intensity
n=2
n=1
n=2
n=1
bioUFM1_1
bioUFM1_2
Intensity vs. presence in 1 or 2 replicas

**Supplementary Figure S6. Analysis of the Mass Spectrometry data for bioUFM1 conjugates in mammalian cells.** A. Venn diagram showing the overlap existing between the total number of proteins identified in cells expressing *bioUFM1-UFC1* (bioUFM1) *versus BirA-puro* (BirA). B. Frequency histograms for bioSmt3 and BirA replicates indicating the number of proteins (*y* axis) found within different range of intensities (*x* axis) in MS analysis. Proteins for which no intensity was recorded were depicted in the lowest range of each group, colored with pink. Intensities are represented in $\log_2$ scale. C. UFMylated proteins (green) were selected based on a 4-fold intensity ratio between the experimental samples (bioUFM1) and the controls (BirA). Ratios with proteins for which no intensity was available were calculated using the lowest intensity reported in the MS analysis, although they were not represented in the graph. Fold changes (in log2 scale) *versus* the sum of the intensities (in log10 scale) detected among all BirA and bioUFM1 analysis are plotted. D. Number of proteins classified as UFM1-conjugates after subtraction of those considered background (i.e. bioSmt3/BirA intensity ratio <4). E. Overlapping degree of the UFM1 conjugates identified between the two replicates (bioUFM1_1 versus bioUFM1_2). F. Reproducibility between MS replicas. The Scatter plot represents the protein intensities (in log2 scale) obtained from replica 1 (bioUFM1_1) *versus* intensities from replica 2 (bioUFM1_2). The $r^2$ statistic was only calculated for proteins with an intensity value in both replicas ($r^2 = 0.7856$). Proteins with intensity only in one replica were also plotted (red and blue dots). In this case missing intensities were estimated to allow graphical representation. G. More abundant proteins have better chances to be detected in both replicas than less abundant ones. Proteins detected in two replicas (n=2) are those found with bigger intensity (in $\log_2$ scale), while those detected in one replica (n=1) present lower intensity.

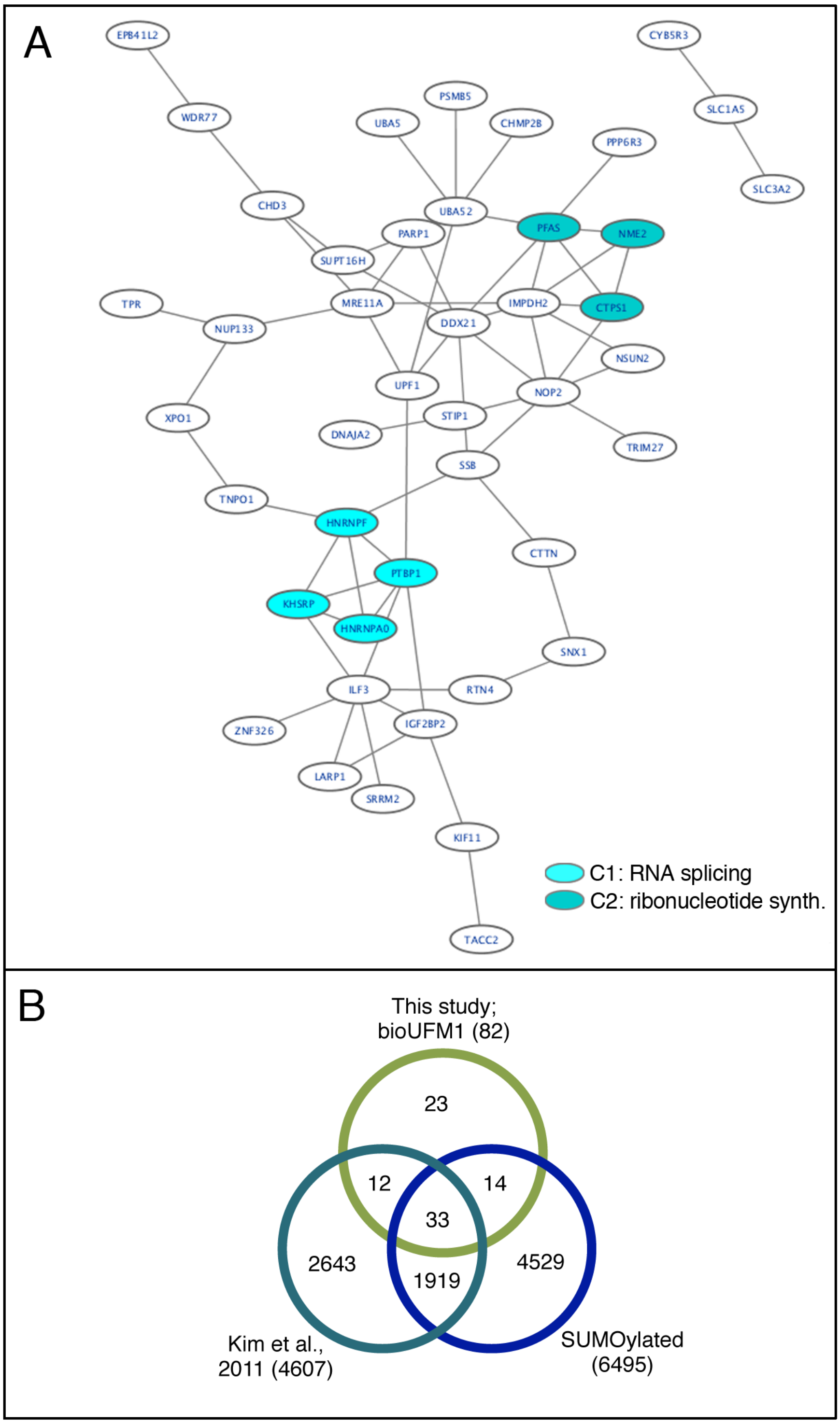


**Supplementary Figure S7. Networking analysis for bioUFM1 conjugates.** A. Cytoscape analysis of the bioUFM1 network. Ovals represent the different UFMylated proteins. Clusters of proteins detailed in Supplementary Data S4 are colored according to the legend. B. Venn diagrams showing the overlap of the bioUFM1 positive hits with reported proteins modified by SUMO or Ub (Kim et al., 2011; right).

**Supplementary Data S1. Composition of the bioUbL vectors generated for this study.**

**Supplementary Data S2. Mass spectrometry results and analysis of bioSmt3 conjugates in *Drosophila* S2R+ cultured cells.**

**Supplementary Data S3. Mass spectrometry results and analysis of bioSmt3 conjugates in *Drosophila* larvae.**

**Supplementary Data S4. Mass spectrometry results and analysis of bioUFM1 conjugates in HEK 293FT cultured cells.**
